# An RBPMS-driven splicing regulatory axis including MBNL1, RBFOX2, and QK promotes smooth muscle cell contractile identity

**DOI:** 10.1093/nar/gkaf1386

**Published:** 2025-12-22

**Authors:** Yuling Huang, Rafael Kollyfas, Ruth Partridge, Clare Gooding, Sanjay Sinha, Irina Mohorianu, Aishwarya Jacob, Christopher W J Smith

**Affiliations:** Department of Biochemistry, University of Cambridge, Cambridge, CB2 1QW, United Kingdom; MRC-Wellcome Cambridge Stem Cell Institute, University of Cambridge, Cambridge, CB2 0AW, United Kingdom; Department of Biochemistry, University of Cambridge, Cambridge, CB2 1QW, United Kingdom; Department of Biochemistry, University of Cambridge, Cambridge, CB2 1QW, United Kingdom; MRC-Wellcome Cambridge Stem Cell Institute, University of Cambridge, Cambridge, CB2 0AW, United Kingdom; MRC-Wellcome Cambridge Stem Cell Institute, University of Cambridge, Cambridge, CB2 0AW, United Kingdom; Department of Biochemistry, University of Cambridge, Cambridge, CB2 1QW, United Kingdom; MRC-Wellcome Cambridge Stem Cell Institute, University of Cambridge, Cambridge, CB2 0AW, United Kingdom; Department of Biochemistry, University of Cambridge, Cambridge, CB2 1QW, United Kingdom

## Abstract

Phenotype switching of vascular smooth muscle cells (SMCs) between contractile and more proliferative and motile states is associated with cardiovascular disease and is underpinned by transcriptional and alternative splicing (AS) programs. We previously showed the RNA-binding protein (RBP) RNA Binding Protein with Multiple Splicing (RBPMS) to be a master regulator of AS in differentiated SMCs. Although changes in master regulator activities can drive AS programs, such proteins rarely act alone. Here we investigated how MBNL1, RBFOX2, and QK act as coregulators with RBPMS to promote contractile smooth muscle AS and phenotypic properties. All four RBPs largely promoted contractile phenotype splicing, with RBPMS showing the highest degree of alignment with the program. Coregulated splicing events were enriched for functions associated with actin filaments and focal adhesions indicating RBPMS-coordinated remodelling of the cellular contractile and motility machinery. Strikingly, while knockdown of each RBP affected various cell morphological and functional properties, knockdown of RBPMS alone induced all aspects of phenotype switching, including lower contraction, higher proliferation, and motility. Our results highlight how a master regulatory RBP can guide an axis of more widely expressed regulators to drive key cellular phenotype changes independently of a transcriptional program.

## Graphical Abstract

**Graphical Abstract:**
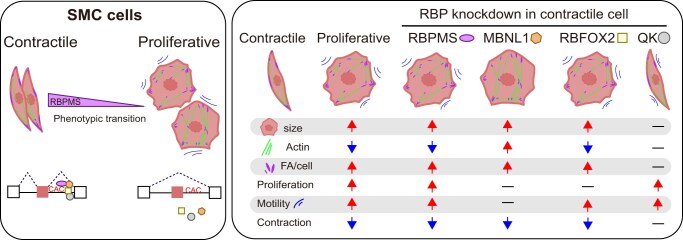

## Introduction

**Figure 1.**
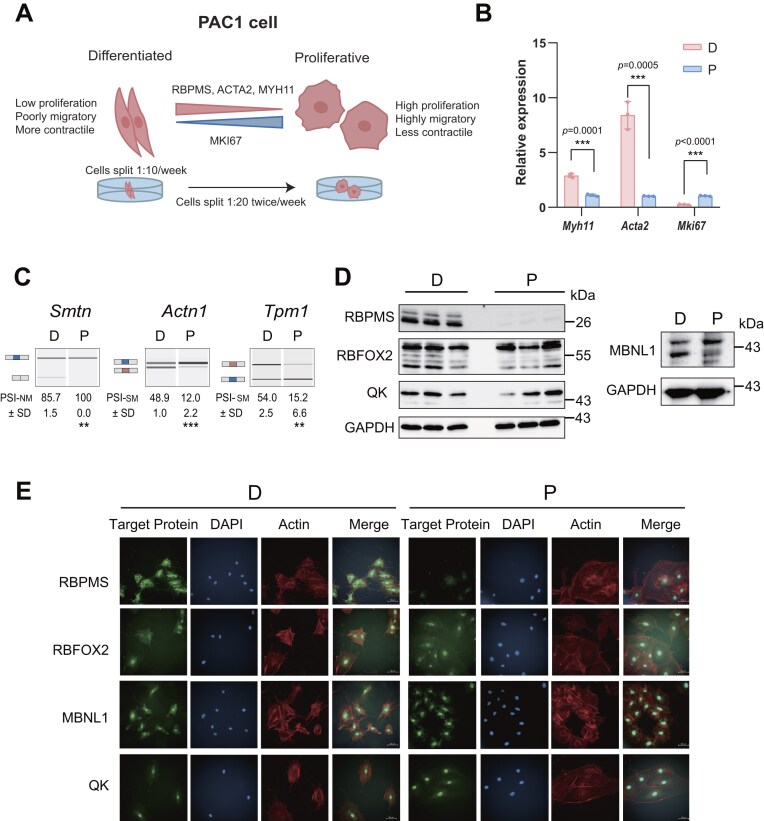
RBPMS is predominantly expressed in differentiated PAC1 cells, while other RNA-binding proteins (RBPs) are expressed in both phenotypes. **(A)** Schematic model illustrating the phenotypic differences between SMCs and the maintenance of differentiated (D) and proliferative (P) states in the PAC1 cell line. Note that before this work the differences in proliferation, migration, and contraction had not been demonstrated. **(B)** Bar plot shows relative messenger RNA (mRNA) expression of Myh11, Acta2, and Mki67 in D and P PAC1 cells, quantified by reverse transcription and quantitation polymerase chain reaction (RT-qPCR) and normalized to CanX. Data represent mean ± standard deviation (SD), *n* = 3. Statistical significance was assessed using unpaired two-tailed *t*-tests. **(C)** Reverse transcriptase-polymerase chain reaction (RT-PCR) analysis of representative AS events (ASEs) in Smtn, Actn1, and Tpm1 in D and P PAC1 cells. Percent spliced in (PSI) values (mean ± SD, *n* = 3) are shown below each image. Schematic diagrams depict the nonmuscle (blue) and smooth muscle (pink) isoforms. Statistical comparisons were made using unpaired two-tailed *t*-tests. **(D)** Western blot of RBPMS, RBFOX2, QK, and MBNL1 protein levels in D and P PAC1 cells. GAPDH serves as a loading control. Lanes 1–3 and 4–6 represent biological replicates. **(E)** Immunofluorescence images showing subcellular localization of RBPMS, RBFOX2, MBNL1, and QK in D and P PAC1 cells. Target proteins (green, specific antibody), actin filaments (red; phalloidin), and nuclei (blue; 4′,6-diamidino-2-phenylindole (DAPI)) are shown. Scale bars, 50 µm.

Smooth muscle cells (SMCs) are important components of multiple tissues, typically residing in the walls of hollow organs such as blood vessels, the alimentary canal and urinary bladder. Contraction of SMCs can therefore influence blood flow and pressure, movement of materials along the gut and of urine from the kidney via the bladder. SMCs differ from striated muscle cells (cardiac and skeletal) in a number of respects. In particular, SMCs are not terminally differentiated and retain phenotypic plasticity (Fig. 1A) [1, 2]. Vascular SMCs (VSMCs), for example, can switch from the mature contractile phenotype typical of a healthy vessel wall, to a more proliferative, motile phenotype in response to injury to the vessel wall or development of atherosclerotic plaques [2–4]. The switch in VSMC phenotypes is driven by a gene expression program with transcriptional and post-transcriptional components. Transcription factors such as Serum Response Factor and the master transcriptional co-activator Myocardin drive expression of genes, such as *Myh11, Smtn*, and *Myl9*, that are necessary, but not sufficient, for the contractile phenotype [5]. A program of alternative splicing (AS) between cell phenotypes has a particular focus upon components of the cell adhesion, and actomyosin contractile and cytoskeletal machineries [5–7]. Notably, the AS program also generates a SMC specific isoform of MYOCD [8, 9], while MYOCD itself helps to drive expression of the SMC master splicing regulator RNA Binding Protein with Multiple Splicing (RBPMS) [10] leading to mutual reinforcement between the transcriptional and post-transcriptional arms of the differentiation program.

**Figure 2.**
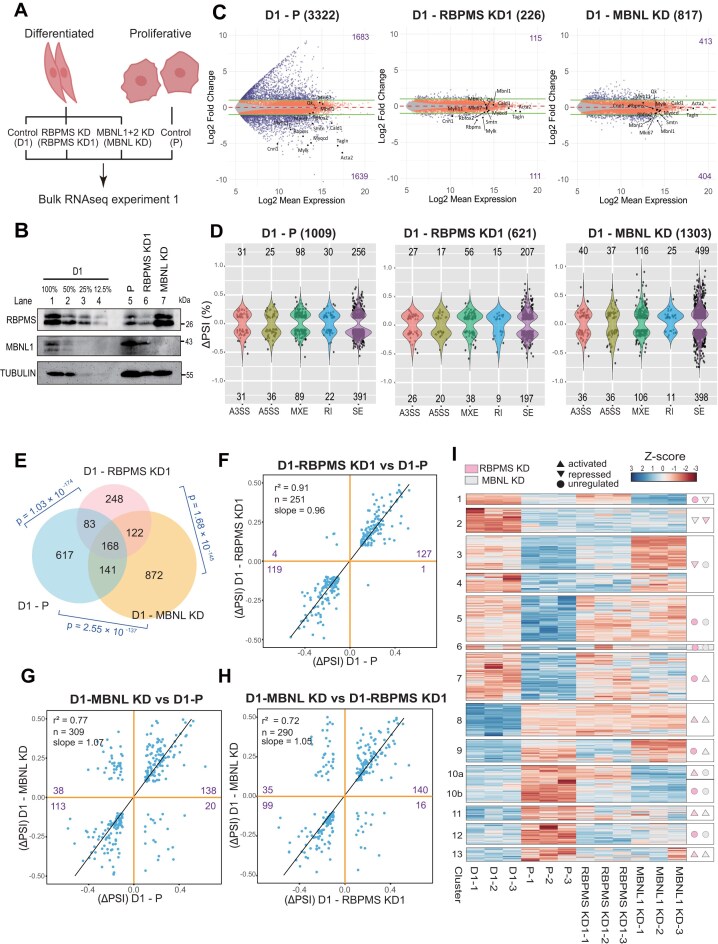
RBPMS and MBNL1 co-regulate ASEs associated with the differentiated SMC phenotype. **(A)** Schematic of the RNA-seq experiment 1 design. Differentiated PAC1 cells (D1) were treated with small interfering RNAs (siRNAs) targeting RBPMS (RBPMS KD1) or MBNL1 and MBNL2 (MBNL KD) for a total of 120 h using a two-hit transfection protocol (second transfection at 48 h). Proliferative PAC1 cells (P) treated with control siRNA were included to compare RBP-regulated splicing events with the endogenous splicing program during SMC phenotypic switching. **(B)** Western blot validation of knockdown efficiency for RBPMS and MBNL1 in D1 cells, along with their endogenous expression in P cells. TUBULIN was used as a loading control, and molecular weights are indicated to the right of each blot. Lanes 1–4 have a titration of D1 control cell sample to allow assessment of knockdown efficiency in lanes 6 and 7. **(C)** MA plots showing differential gene expression between (left to right): D1 versus P, D1 versus RBPMS KD1, and D1 versus MBNL KD. Genes with significant changes in mRNA abundance (adjusted *P* <.05) are shown in red. Grey dots indicate genes without significant expression changes. Horizontal dashed lines represent log_2_ fold changes of +1 and −1. Genes with significant changes that exceed these thresholds are highlighted in dark blue. Selected marker genes are annotated. Purple numbers indicate the number of significantly upregulated and downregulated genes. **(D)** Violin plots showing the distribution of ASE types—including alternative 3′ splice site (A3SS), alternative 5′ splice site (A5SS), mutually exclusive exon (MXE), retained intron (RI), and skipped exon (SE)—in each comparison: D1 versus P, D1 versus RBPMS KD1, and D1 versus MBNL KD. The number of significant events [junction count ≥ 50, |ΔPSI| ≥ 0.1, false discovery rate (FDR) < 0.05] is indicated above each plot. **(E)** Venn diagram showing the overlap of significant splicing events identified in the D1 versus P, D1 versus RBPMS KD1, and D1 versus MBNL KD comparisons. The statistical significance of pairwise overlaps was assessed using the hypergeometric test, and *P*-values are indicated in blue. **(F–H)** Scatter plots comparing ΔPSI values between conditions: **(F)** D1-RBPMS KD1 versus D1-P, **(G)** D1-MBNL KD versus D1-P, and **(H)** D1-MBNL KD versus D1-RBPMS KD1. Pearson correlation coefficients (*r²*) and linear regression slopes are indicated for coregulated events (upper right and lower left quadrants). Numbers of ASEs in each quadrant are shown in purple, *n* = number of splicing events. **(I)** Heatmap showing 663 ASEs that are regulated during PAC1 cell differentiation, defined from the D1 control versus P control comparison. PSI Z-scores were row-normalized, and hierarchical clustering was performed across all conditions. Each row represents one splicing event. Arrows denote the inferred regulatory action of RBPs on each event: activation (▲), repression (▼), or unregulated (°).

While AS can be regulated by the chromatin environment, RNA Pol II elongation [11] and changes in activity of core splicing factors [12], AS programs are largely regulated by auxiliary RBPs [13]. While some RBPs are widely expressed, with unchanged activities during phenotype transitions, others show more restricted expression and can act as “master regulators” that are sufficient to induce specific AS programs [14]. RBPMS is such a master splicing regulator in VSMCs. RNAi-mediated depletion in differentiated PAC1 VSMCs showed that changes in RBPMS expression alone are sufficient to fully account for at least 20% of the AS program between differentiated and proliferative cells [7]. Likewise, induced overexpression of RBPMS in either proliferative PAC1 cells or human VSMCs differentiated from embryonic stem cells (hES-VSMCs) induces numerous SMC-specific splicing patterns, including some that are usually observed only in fully differentiated mature VSMCs *in vivo* [7, 15]. In hES-VSMCs, RBPMS overexpression reduced motility and proliferation, consistent with induction of a more contractile phenotype [15]. Homozygous *Rbpms* null mice show overproliferation of VSMCs during embryonic development [16], while RBPMS knockout and overexpression in adult VSMCs showed reciprocal effects upon vascular remodelling that are consistent with RBPMS playing an important role in promoting the VSMC contractile state [17]. Indeed, single cell RNA-Seq profiling of tissue VSMCs has shown *Rbpms* to be part of the contractile VSMC transcriptional signature [15, 17, 18].

Master splicing regulators are not expected to act independently of other regulatory RBPs [14]; rather, for specific coregulated programs of ASEs, the master regulator may be the single regulatory component that is needed to “flick the switch”. Indeed, we previously found that via its C-terminal intrinsically disordered region (IDR), RBPMS is able to interact with numerous other RBPs and splicing factors [19]. Among these, MBNL1 and RBFOX2 co-regulated a panel of 11 candidate ASEs with RBPMS [19]. Additional evidence for collaboration between RBPMS, MBNL1, and RBFOX2 in VSMCs comes from the AS changes induced by overexpression of MYOCD [10], and the significant overlap of ASEs regulated by overexpression of RBPMS and knockdown of RBFOX2 in hES-VSMCs [15]. Another RBP that has been shown to regulate VSMC phenotype, but in the opposing direction, is Quaking (QK) [9], which promotes skipping of *Myocd* exon 2a to generate the cardiac specific isoform [8]. In contrast, RBPMS promotes *Myocd* exon 2a inclusion and also of the H1 exon of *Flnb* [7], which is skipped under the influence of QK in breast cancer cells [20].

Here we set out to explore the global contributions of RBPMS, MBNL1, RBFOX2, and QK in controlling the transcriptome and functional phenotypes of differentiated PAC1 VSMCs. We hypothesized that RBPMS, MBNL, and RBFOX2 proteins would act collaboratively to promote a differentiated program, while QK would act antagonistically to promote a proliferative program. Knockdown RBPs followed by RNA-Seq showed that all four RBPs, including QK, regulated SMC specific ASEs overwhelmingly in the same direction, promoting splicing patterns associated with the differentiated phenotype, particularly among genes encoding actin cytoskeletal/contractile and cell adhesion machineries. Many ASEs were coregulated by three or four of the RBPs and analysis of binding motifs associated with each protein showed that their cognate motifs were closely spaced adjacent to regulated exons, suggesting direct collaborative regulation of target exons. Indeed, all three coregulators co-immunoprecipitated with RBPMS and these interactions depended upon functionally important aromatic residues in RBPMS, and the interaction with RBFOX2 also depended upon functionally important tyrosine residues. Knockdown of all RBPs affected various characteristics of differentiated SMCs, including cell morphology, actin architecture, focal adhesion (FA) number, proliferation, motility, and contraction. Strikingly, knockdown of RBPMS alone shifted all observed properties from the contractile towards the proliferative phenotype, consistent with its closer alignment with the differentiation splicing program. These phenotypic changes in the absence of major changes in the transcriptional program underline the importance of the RBPMS regulatory axis in promoting the differentiated contractile phenotype via a program of regulated AS.

## Materials and methods

### Cell culture and transfection

Rat PAC1 pulmonary artery SMCs [21] were cultured under conditions that promote either a differentiated (PAC1-D) or a proliferative (PAC1-P) gene expression program according to the protocol described previously [6] (Fig. 1A). HEK293T cells were passaged twice per week at a 1:20 dilution. HEK293T cells were transfected using Lipofectamine 2000 (Thermo Fisher) according to the manufacturer’s protocol. For overexpression, 3 × 10⁵ cells were seeded per well in six-well plates and transfected with 0.4–2 µg plasmid DNA in Opti-MEM. To maintain a constant total DNA concentration across isoforms and mutants, empty vector DNA was added.

For siRNA-mediated knockdown in PAC1 cells, reverse transfection was used with 90 pmol siRNA in total and 8 µl Lipofectamine RNAiMAX (Thermo Fisher). PAC1-D and PAC1-P cells were seeded at 3.5 × 10⁵ and 2.5 × 10⁵ per well in six-well plates, respectively. Morphological and phenotypic changes in PAC1 cells following RBP knockdown were assessed following a two-hit siRNA mediated knockdown with the second siRNA transfection following 48 h after the initial transfection, and final assessment of cells 120 h after the first knockdown. A scrambled siRNA sequence, referred to as C2, was used as a negative control in all knockdown experiments. All siRNA sequences are tabulated in Supplementary File 1. All siRNAs for RBPMS, MBNL1, MBNL2, RBFOX2, and QK have been previously validated [6, 7, 19, 22]. Each condition was repeated in at least three biological replicates. For pInducer-expressing PAC1 cells [7], doxycycline (1 µg/ml) was added 24 h after siRNA treatment to induce 3xFLAG-RBPMSA expression. Cells were harvested 24 h after induction. The transfections were verified by western blot (antibodies used in Supplementary File 1).

### RNA extraction and complementary DNA synthesis

Total RNA was extracted using TRI Reagent (Sigma) following phase separation with chloroform and isopropanol precipitation. RNA was treated and inactivated with TURBO™ DNase (Invitrogen). RNA quality and concentration were assessed using a NanoDrop spectrophotometer (Thermo Scientific). For RNA-seq experiments, total RNA was purified using the Direct-zol RNA Miniprep Kit (Zymo Research), including on-column DNase digestion. For RT-PCR and qPCR analyses, 1 µg of DNase-free RNA was reverse transcribed using Reverse Transcriptase (Roche) and oligo-dT primers according to the manufacturer’s instructions.

### DNA constructs

Rat RBPMS-A (NCBI accession number: *XM_006253240.2*) cloned into the pEGFP-C1 vector (Clontech) and human MBNL1 isoform a (amino acids 1–382, NCBI accession number: *NP_066 368*) fused to EGFP at the N-terminus were described previously [7, 22, 23]. Full-length RBFOX2 (*NM_001414967.1*) and QK5 (*NM_001414465.1*) isoforms from *Rattus norvegicus* were amplified by RT-PCR from PAC1 cell complementary DNA (cDNA) using isoform-specific primers (Supplementary File 1) and initially cloned into the pGEM-T Easy vector (Promega) for sequence verification. To generate tagged constructs for expression studies, RBFOX2 fragments were subcloned into pCI-neo-V5-TEV using NheI and EcoRI sites. QK5 was cloned into pCI-neo-MYC using AvrII and MluI. The original FLAG tag in pCI-neo was replaced by a V5-TEV tag via NheI and BamHI. All oligonucleotide sequences are in Supplementary File 1. The FLAG-RBPMS-WWF + 3YW mutant was generated by site-directed mutagenesis of tyrosines 141, 151, and 154, tryptophans 148, 180, and 191, and phenylalanine 196 to alanine (see Supplementary Fig. 6). The FLAG-RBPMS-ΔC mutant lacks amino acids 114–197. The RBFOX2-10YS mutant constructs were generated using the NEBuilder^®^ HiFi DNA Assembly Kit (New England Biolabs) in a four-fragment assembly. Fragments 1 and 4 of RBFOX2 were PCR-amplified from the V5-TEV-RBFOX2 construct, whereas the 10YS mutant region (fragments 2 and 3) was synthesized by Sigma.

### Co-immunoprecipitation and western blots

Co-immunoprecipitation (co-IP) was performed using 1 mg of total protein lysate prepared in radioimmunoprecipitation (RIPA) buffer [150 mM NaCl, 1% Triton X-100, 0.1% sodium dodecyl sulphate (SDS), 50 mM Tris, pH 7.5] supplemented with protease inhibitors (Roche), benzonase (Millipore), and 1 mM phenylmethylsulfonyl fluoride (PMSF). Lysates were pre-cleared and incubated overnight at 4°C with 3 µg of anti-Flag primary antibody. The following day, 40 µl of Protein G Dynabeads (Invitrogen) was added and incubated at room temperature for 1 h with gentle rotation. Bead-bound complexes were washed 3 × with phosphate buffered saline with 0.1% Tween 20 (PBST) and eluted with 0.1 M glycine (pH 2.5), followed by neutralization with 1 M Tris (pH 7.5). Samples were mixed with 6 × SDS loading buffer, denatured at 95°C for 5 min, and analysed by sodium dodecyl sulphate–polyacrylamide gel electrophoresis (SDS–PAGE) and western blot.

For western blots, protein samples were mixed with either 2 × or 6 × SDS loading dye and denatured at 95°C for 5 min. Proteins were resolved by SDS–PAGE and transferred onto Immobilon-FL polyvinylidene difluoride (PVDF) membranes (Millipore). Membranes were blocked with 5% milk or bovine serum albumin (BSA) in TBST for 1 h at room temperature, incubated with primary antibodies overnight at 4°C, and then with appropriate secondary antibodies. Signals were detected using the Clarity or Clarity Max ECL system (Bio-Rad) and visualized using a ChemiDoc imaging system (Bio-Rad). Details of the antibodies used are provided in Supplementary File 1.

### qRT-PCR and RT-PCR

Quantitative PCR was performed using SYBR JumpStart Taq ReadyMix (Sigma) on a Rotor-Gene Q system (Qiagen), with gene-specific primers (Supplementary File 1). Each target gene was analysed in technical triplicates. Relative expression levels were normalized to housekeeping genes *Gapdh* and *Canx*. No-template controls were included to ensure specificity. Comparative quantification was calculated using Rotor-Gene Q Software 1.7. Expression values were acquired from three biological repeats. Alternative exon inclusion was assessed by RT-PCR using 50 ng of cDNA and primers listed in Supplementary File 1. Products were resolved using the QIAxcel system, and PSI values were quantified with ScreenGel software. Data are reported as mean (%) ± SD, with statistical analysis performed using an unpaired Student’s *t*-test or two-way analysis of variance (ANOVA).

### Immunostaining and proliferation assay

To assess the influence of RBP depletion on cell morphology and proliferation, control and RBP knockdown PAC1 cells were cultured on glass coverslips for 24 h following the final siRNA treatment or after EdU labeling (see below). For immunodetection of RBP localization, cells were grown on glass coverslips for 24 h, fixed in 4% paraformaldehyde for 10 min, permeabilized with 0.1% Triton X-100 for 10 min, and blocked with 5% BSA in PBST for 1 h. Primary antibodies were incubated for 1 h at room temperature or overnight at 4°C, followed by appropriate fluorescent secondary antibodies for 1 h. Nuclei were counterstained with DAPI, and F-actin was visualized using Alexa Fluor 488 or 594 phalloidin (Invitrogen). To detect the effects of RBP knockdown on SMC proliferation, cells were seeded on glass coverslips and cultured for 24 h after siRNA treatment, followed by incubation with 10 µM EdU for 12 h. EdU incorporation was detected using the Click-iT™ EdU Cell Proliferation Kit for Imaging (Invitrogen). Microscopy imaging was performed to visualize EdU-positive cells. Fluorescence images were acquired using either a Zeiss Axio Imager Z2 microscope equipped with an AxioCam HRm CCD camera and Plan-Apochromat 40×/1.3 oil immersion objective, or a Nikon C2 laser-scanning confocal microscope with a 40×/1.3 oil objective. For confocal imaging (Nikon C2), laser excitation lines at 405, 488, 561, and 640 nm were used for DAPI, Alexa Fluor 488, Alexa Fluor 594, and Alexa Fluor 647 respectively. Emission detection windows were 425–480 nm, 500–550 nm, and 580–620 nm. Image analysis was performed using Fiji (ImageJ v1.54p). Uniform linear brightness and contrast adjustments were applied across the entire field without altering raw intensity values. For cell size quantification, phalloidin-stained actin images were used. Individual cell boundaries were manually traced using the freehand selection tool, and areas were measured via the Region of Interest (ROI) Manager. The same manually defined ROIs were reused for quantifying actin fiber anisotropy using the FibrilTool ImageJ/Fiji macro (v1.0) [24], which computes the degree of cytoskeletal alignment within each cell. Nucleus size was measured by thresholding the DAPI channel and analysing individual nuclear masks. FA were visualized by paxillin staining. A binary FA mask was generated from the paxillin channel. The total number of FAs per cell were analysed using the wand tracing tool in conjunction with the ROI Manager in ImageJ.

For each condition, ≥20 cells were analysed per experiment. A total of three independent experiments were performed, and each experiment included three replicates derived from separate culture plates. Statistical significance was evaluated using unpaired Mann–Whitney tests.

### Cell motility

Cell migration was assessed using an ibidi Culture-Insert two-well placed in a µ-Dish, which created a 500 µm cell-free gap. Twenty-four hours after the final siRNA treatment, 3 × 10^4^ cells were seeded into each well of the insert and cultured until confluence. The inserts were then gently removed with sterile tweezers, the gaps rinsed with pre-warmed phosphate buffered saline, and fresh Dulbecco’s modified Eagle’s medium was added. Phase-contrast images were captured every 60 min over an 9-h period. Cell front progression was analysed using ImageJ, and the migration speed was calculated as the cell front velocity (µm/h) by tracking the forward movement of the leading edge over time. Only the linear phase of migration was used for speed calculation, and analysis was restricted to the first 8 h to minimize confounding effects from proliferation. All experiments were performed in three biological replicates, and statistical significance between groups was assessed using Student’s *t*-test.

### Contraction assay

Knockdown and control PAC1 cells were seeded onto glass-bottom 24-well plates and allowed to adhere for 24 h. Baseline cell spread areas were imaged in predefined fields using either an Opera Phenix System (PerkinElmer) equipped with a 20 × water objective, or a Leica THUNDER Imager 3D Cell Culture system with a 20 × dry objective under transmitted light (brightfield). Cells were then treated with 10 µM ionomycin (Invitrogen) for 15 min at 37°C to induce contraction, followed by imaging of the same fields post-treatment. For each field, individual cells with clearly defined boundaries and no visible signs of damage post-treatment were manually selected and analysed. Cell areas were quantified using ImageJ. Cell boundaries were traced with the freehand selection tool and stored in the ROI Manager to measure the cell spread area. Percentage contraction was calculated as the relative reduction in cell area compared to the corresponding baseline measurement of the same cell. Each condition was assessed in at least three independent biological experiments, included three biological replicates, each derived from independently plated. A total of ∼45–70 cells were analysed per condition experiment replicates. Statistical significance was evaluated using unpaired Mann–Whitney tests.

### RNA-seq data generation and preprocessing

RNA-seq was performed on total RNA extracted from siRNA-treated PAC1 cells.Libraries of poly(A)-selected RNA were prepared using the NEBNext Ultra II RNA Library Prep Kit and sequenced on an Illumina NovaSeq 6000 platform (150 bp paired-end reads). Initial quality control was conducted by the sequencing facility.

The quality of the sequencing data was further assessed using FastQC (v0.11.9) on all raw FASTQ files, with outputs summarized using MultiQC (v1.14). Initial sequencing depths ranged between 56 million and 93.8 million reads per sample. To ensure consistency, subsampling without replacement was performed using seqtk (v1.3-r106), uniformly reducing sequencing depth to 55 million reads across all samples. Subsampled paired-end reads were aligned to the *R. norvegicus* reference genome mRatBN7.2, NCBI RefSeq assembly GCF_015227675.2 (suppressed) using STAR aligner (v2.7.11b) [25]. Resulting BAM files were sorted by genomic coordinates. Gene-level expression quantification was performed using featureCounts (v2.0.8), guided by annotations provided by the NCBI RefSeq rn7 GTF file.

### Noise reduction and differential expression

Noise estimation and reduction were performed on the gene expression count matrix using noisyR (v1.0.0) [26]. Expression values were subsequently normalized using quantile normalization [27]. Differential gene expression analysis was performed independently using edgeR [28] and DESeq2 [29], achieving high convergence post-noise correction. Genes with FDR-adjusted *P*-values <.05 and |log_2_FC| >1 were considered significantly changed.

### AS analysis

AS was assessed and quantified using rMATS (v4.3.0) [30], categorizing splicing events into SE, MXE, A5SS, A3SS, and RI. Quantitation of PSI used junction counts only. Splicing events were filtered to retain those with total junction read counts ≥50 across three replicates for both conditions being compared, FDR < 0.05, and absolute change in percent spliced in (ΔPSI) ≥10%.

### Motif enrichment analysis

Enrichment of RBP motifs was assessed using the Matt toolkit (v1.3.1) [31]. Analyses were performed on sequences spanning ± 250 bp around cassette exons. For each event, sliding window scanning (window size = 31 bp) and exon/intron partitioning were applied. Known binding motifs for RBPMS (CAC-N₃₋₁₅-CAC), RBFOX (GCATG), MBNL (YGCY-N₅₋₂₀-YGCY), and QK (ACTAA or CTAAC) were evaluated. All chosen motifs were validated using mutated versions, as shown in Supplementary Fig. S5 and its legend. All linked motifs were separated by a spacer of 5–25 nucleotides. Cassette exons showing significant AS changes (FDR < 0.05 and a relaxed |ΔPSI| threshold of ≥ 5%) were used as the test set. Unregulated exons were defined as FDR > 0.1 and |ΔPSI| < 5%. Both sets passed the junction read count (≥50) filter. RNA maps were generated using the rna_maps function from the Matt suite. For comparison, the background set of unregulated events was randomly downsampled to 2000 exons. Motif enrichment was analysed within a 135 bp region of the cassette exon adjacent to each splice site and 250 bp of each flanking intronic region. The statistical significance of enrichment or depletion was assessed using permutation tests (*n* = 1000 iterations), with regions considered significant at *P* <.05.

### Gene ontology analysis

Gene ontology (GO) enrichment analysis of differentially expressed genes was performed using g:Profiler [32] using all genes expressed above the noise level as background set. GO enrichment for alternatively spliced genes was performed using Gorilla [33]. Background gene lists for the PAC1 experiments were generated by selecting genes with expression levels above the estimated noise threshold following normalization. The selected biological process, cellular component (CC), and molecular function term are reported. For splicing, only events passing all filters (Junction Counts (JC) ≥ 50, FDR < 0.05, ΔPSI ≥|10%|) were included.

### Statistical analysis

All experiments were conducted with at least three independent biological replicates. Analysis and quantification of RNA-seq, RT-PCR, and imaging experiments are described in their respective sections. Details of the statistical tests used for each experiment are provided in the corresponding figure legends. Graphs were generated using RStudio, GraphPad Prism, and Adobe Illustrator.

### Visualization and interactive exploration

For data visualization and interactive exploration, a shareable Shiny web interface was created using bulkAnalyseR (v1.1.0) [34]. The distribution of sequencing signals across transcripts was visually assessed by loading indexed BAM files into the IGV Browser [35]. Raw and processed RNA-seq data have been deposited in NCBI GEO under accession numbers GSE301925 and GSE210586. The analysis and dataset, through the bulkAnalyseR interface are publicly available at https://mohorianulab.org/shiny/CSmith/Huang2025/.

## Results

Rat PAC1 pulmonary VSMC cells [21] can be cultured under conditions promoting more differentiated or proliferative phenotypes, providing a cell culture model of vascular SMC plasticity (Fig. 1A) [7], as measured by known markers of smooth muscle transcriptional upregulation (Fig. 1B) and AS patterns (Fig. 1C) [2, 5, 7, 36, 37]. To investigate whether RBPMS and its candidate co-regulators coordinate AS during SMC phenotypic transitions, we first examined their expression and subcellular localization in differentiated and proliferative PAC1 cells (referred to subsequently as PAC1-D and PAC1-P cells). RBPMS, RBFOX2, and MBNL1 are the dominantly expressed members of their protein families (RBPMS/RBPMS2, RBFOX1/2/3, MBNL1/2/3) in PAC1 cells [7]. We therefore focused on these family members, although in siRNA-mediated knockdowns we targeted MBNL1 and 2 to prevent compensation by upregulated MBNL2 [22]. Western blots (Fig. 1D) and qRT-PCR (Supplementary Fig. S1) showed expression of RBFOX2, QK, and MBNL1 at similar levels in PAC1-D and P cells, although MBNL1 showed a shift in protein isoforms (Fig. 1D) as observed previously [7]. RBPMS alone was substantially downregulated between PAC1 D and P cells (Fig. 1D). All four RBPs localize primarily to the nucleus (Fig. 1E), although a fraction of RBPMS was also present in the cytoplasm of PAC1-D cells. Again, RBPMS was the only RBP substantially downregulated in proliferative cells. The even expression of RBFOX2, MBNL1, and QK between PAC1 phenotypes implies that these factors are unlikely to drive phenotypic change. Nevertheless, they may be essential coregulators with RBPMS, whose expression level changes are the primary driver of a differentiated SMC post-transcriptional program.

To compare the global contributions of the four RBPs to the differentiated SMC transcriptome, we carried out siRNA-mediated knockdown followed by RNA-Seq. For practical reasons two separate RNA-Seq experiments were carried out. In one experiment, we knocked down RBPMS and MBNL1/2 in PAC1-D cells (Fig. 2A), and in parallel PAC1 D and P cells were treated with control siRNA to define the reference transcriptomic and splicing landscapes of SMC differentiation. Western blots confirmed the knockdown of RBPMS and MBNL1 (Fig. 2B). Following RNA-Seq, changes in RNA abundance were analysed by DESeq2 (FDR < 0.05, log_2_FC > 1) and splicing changes by rMATS (FDR < 0.05, |ΔPSI| ≥ 10%, junction read counts ≥50) (Supplementary File 2). At the RNA abundance level, the differentiation program (D-P) showed 3322 genes significantly changing (Fig. 2C) reflecting the transcriptional program associated with phenotypic plasticity. Knockdown of RBPMS or MBNL1 showed many fewer mRNA abundance changes (226 and 817, respectively). Among the most substantially downregulated genes in PAC1-P cells were contractile markers (e.g. *Myh11, Acta2, Mylk, Myl9, Cnn1, Smtn, Tagln*) the master transcriptional co-activator *Myocd* and the splicing regulator *Rbpms*. Notably, all these markers were also significantly (FDR < 0.05) downregulated upon RBPMS knockdown. However, the magnitude of changes were far smaller than in the PAC1-D versus P comparison, in some cases not passing the two-fold change threshold. For example, A*cta2* and *Mylk* were downregulated 78- and 38-fold by dedifferentiation but by only 1.7- and 3.0-fold upon RBPMS knockdown. This suggests that RBPMS plays a minor, albeit statistically significant, role in controlling the PAC1 D mRNA abundance program. In contrast, upon MBNL1/2 knockdown differentiation markers changed abundance in both directions, with none passing the fold-change threshold.

The PAC1 differentiation splicing program comprised 1009 ASEs (Fig. 2D). In contrast to the modest impacts on RNA abundance, knockdown of both RBPMS and MBNL led to comparable numbers of splicing changes (621 and 1303, respectively; Fig. 2D). Significantly regulated ASEs showed a high degree of overlap (Fig. 2E) and pairwise comparisons (Fig. 2F–H) between knockdowns and differentiation showed the majority of changes to be congruent, with RBP knockdowns promoting proliferative splicing patterns. Differentiation-specific ASEs regulated by RBPMS, representing 25% of the differentiation program were 98% congruently regulated (nearly all events in upper right and lower left quadrant of Fig. 2F) with a very high correlation between RBPMS knockdown and dedifferentiation (Fig. 2F, r^2^ = 0.91). Notably, RBPMS-regulated events included those with the highest ΔPSI changes during differentiation; 28 of the top 34 differentiation regulated ASEs (|ΔPSI| > 0.4) were co-regulated by RBPMS. The similar magnitudes of ΔPSI upon RBPMS knockdown and dedifferentiation (Fig. 2F, slope = 0.96) indicate that the change in RBPMS expression levels alone are fully responsible for changes in these ASEs between D and P cells [7]. This contrasts with the modest reductions in transcription marker expression upon RBPMS depletion. MBNL1/2 knockdown also regulated differentiation specific ASEs, showing a good correlation with differentiation regulated ASEs for congruently regulated events (r^2^ = 0.77, slope = 1.07, Fig. 2G). However, MBNL1/2 knockdown produced a substantial minority of events (19%) acting in the opposite direction to differentiation, ten-fold more than RBPMS (Fig. 2G, lower right and upper left quadrants). Similarly, ASEs regulated by RBPMS and MBNL1/2 showed mostly congruent changes (r^2^ = 0.72) with 18% of events showing antagonistic regulation (Fig. 2H). Hierarchical clustering of ASEs regulated between PAC1 D and P cells, revealed substantial groups that were congruently coregulated by RBPMS and MBNL1/2, involving both repression of proliferation specific exons and activation of differentiation specific exons (Fig. 2I).

**Figure 3.**
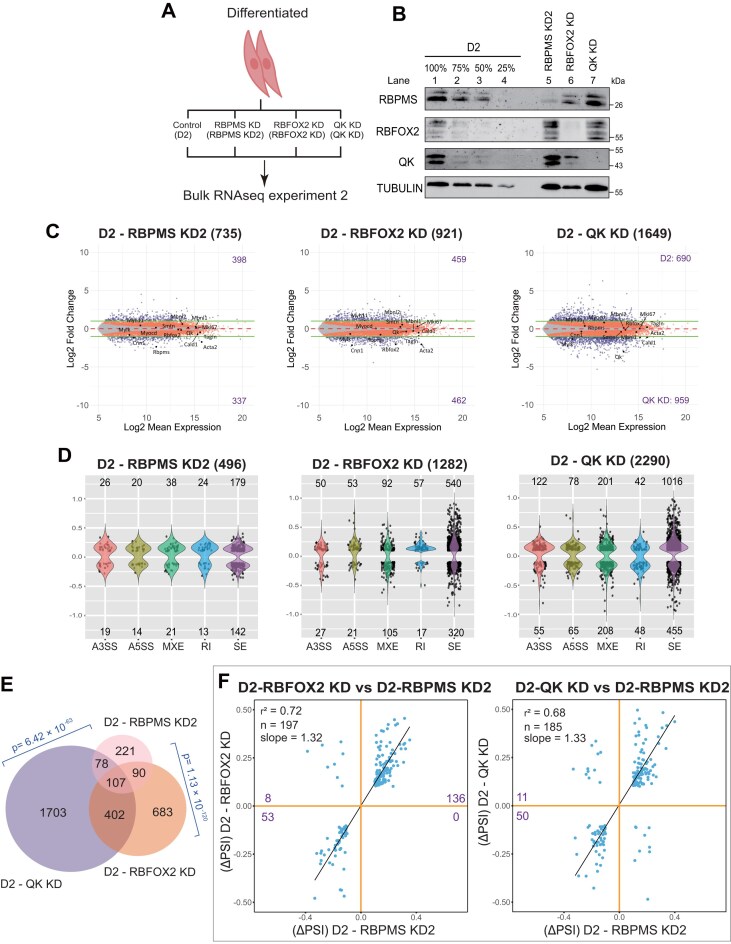
RBFOX2 and QK co-regulate ASEs overlapping with the RBPMS-dependent programme in differentiated PAC1 cells. **(A)** Schematic of RNA-seq experiment 2, showing siRNA knockdown of RBPMS (RBPMS KD2), RBFOX2 (RBFOX2 KD), and QK (QK KD) in differentiated (D2) PAC1 cells. Knockdown was performed using two sequential siRNA transfections, with the second transfection at 48 h and cell harvesting at 120 h after the initial treatment. **(B)** Western blot validation of knockdown for RBPMS, RBFOX2, and QK in D2 PAC1 cells. Lanes 1–4 have a titration of D1 control cell sample to allow assessment of knockdown efficiency in lanes 6 and 7. Tubulin was used as a loading control, and molecular weights are indicated on the right. **(C)** MA plots showing differential gene expression between D2 and RBPMS KD2 (left), RBFOX2 KD (middle), and QK KD (right). Genes with adjusted *P* <.05 are highlighted in red. Genes exceeding log_2_ fold change thresholds of ± 1 are shown in dark blue. Selected marker genes are annotated. Purple numbers indicate the counts of significantly upregulated and downregulated genes. **(D)** Violin plots showing distributions of ΔPSI values across five ASE types for each comparison: D2 versus RBPMS KD2, D2 versus RBFOX2 KD, and D2 versus QK KD. The number of significant events (junction count ≥ 50, |ΔPSI| ≥ 0.1, FDR < 0.05) is indicated above each group. **(E)** Venn diagram showing the overlap of significant splicing events among D2 versus RBPMS KD2, RBFOX2 KD, and QK KD comparisons. Pairwise overlap significance was evaluated using the hypergeometric test, with corresponding *P*-values shown in blue. (**F, G**) Scatter plots comparing ΔPSI values between **(F)** D2-RBFOX2 KD versus D2-RBPMS KD2, and **(G)** D2-QK KD versus D2-RBPMS KD2. The Pearson correlation coefficient (r²) and slope are calculated based on co-regulated events, while the number of splicing events (*n*) and quadrant counts are based on all events. ASEs co-regulated by both RBPs are found in the upper-right and lower-left quadrants.

To directly compare the roles of RBFOX2 and QK with RBPMS in shaping the PAC1 cell transcriptome each protein was knocked down in PAC1 D cells in a second RNA-Seq experiment (Fig. 3A and B). At the RNA abundance level, many more changes were seen upon RBPMS knockdown than in experiment 1 (735 versus 226) (Fig. 3C), although the magnitude of changes were still far less than in the differentiation program (compare Fig. 3C with Fig. 2C). Comparison of control differentiated conditions showed that PAC1-D cells in experiment 2 were less differentiated than experiment 1 (Supplementary Fig. S2D). Comparison of splicing events regulated by RBPMS knockdown in the two experiments showed a surprisingly low overlap with only 103 events passing the FDR, ΔPSI, and junction count filters in both experiments (Supplementary Fig. S2A). These events showed similar directional responses (r^2^ = 0.73; Supplementary Fig. S2A), but the magnitude of splicing changes in experiment 2 was lower (slope 0.57). Direct comparison of absolute PSIs across the two experiments showed much better agreement between knockdown samples (Supplementary Fig. S2B, r^2^ = 0.78, slope = 0.92) than control differentiated samples (Supplementary Fig. S2C, r^2^ = 0.55, slope = 0.71). This indicates a lower degree of differentiation of PAC1-D cells in experiment 2 at both splicing and transcriptional levels. Nevertheless, experiment 2 provided a rich source of information on ASEs regulated by RBFOX2, QK, and RBPMS.

Depletion of RBFOX2 and QK led to substantially more AS changes than RBPMS (2.5- and 4.6-fold, respectively) (Fig. 3D). However, overlapping significant events (Fig. 3E) were mostly coregulated in the same direction, 96% for RBFOX2/RBPMS and 89% for QK/RBPMS (Fig. 3F). Likewise, for QK and RBFOX2 events that were also regulated by differentiation state, the two RBPs promoted the differentiated splicing pattern in the majority of cases: 83% of RBFOX2 regulated events (Supplementary Fig. 2E) and 63% of QK regulated events (Supplementary Fig. 2F), similar to MBNL1/2. This agreed with our initial working hypothesis for RBFOX2 and MBNL1/2, but not for QK which had been predicted to promote a proliferative AS program [9]. Nevertheless, of the four RBPs QK showed the lowest alignment with differentiation.

### A core set of co-regulated SMC exons

**Figure 4.**
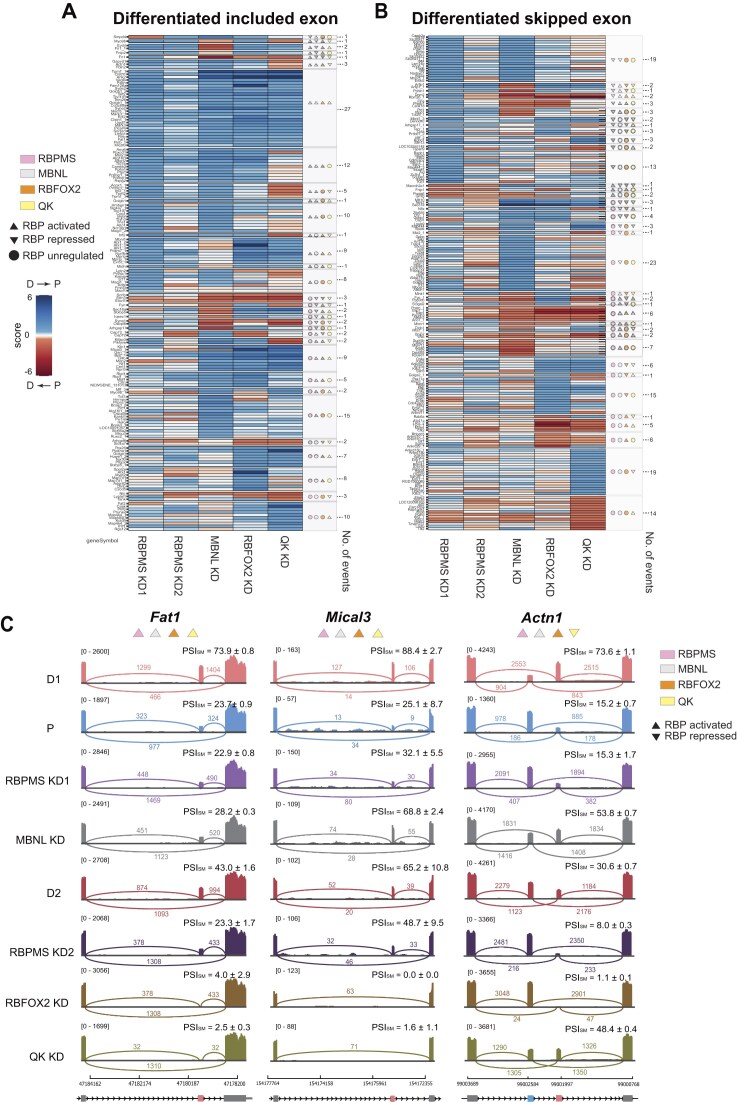
Co-regulation of SMC SE events by four RBPs. **(A, B)** Heatmaps showing the effects of individual RBP knockdowns on SE events that are significantly more included **(A)** or more skipped **(B)** in differentiated (D) versus proliferative (P) PAC1 cells. For each RBP knockdown condition, the ΔPSI change has been normalized to the D versus P ΔPSI from experiment 1. Colours indicate the concordance (blue) or antagonism (red) of RBP activity with the D versus P splicing program. Annotation symbols to the right indicate inferred activity of each RBP: activated (▲), repressed (▼), or unregulated (●). The number on the right of the heatmap indicates the counts of events in each cluster. **(C)** Representative sashimi plots and PSI values (mean ± SD) for three regulated exons-*Fat1, Mical3*, and *Actn1-*corresponding to events shown in the heatmaps above. PSI values and splice junction read counts are shown for each condition (D1, P, D2, and RBP knockdowns). Numbers to the right represent mean PSI values calculated by rMATS. Numbers above the splice junctions indicate supporting read counts. Genome coordinates are shown below each plot. The alternatively spliced exon is shown in pink (smooth muscle exon) and blue (nonmuscle exon), and flanking exons are shown in grey.

To facilitate integration of the contributions of the four RBPs to the PAC1 differentiation program, PAC1 D-P regulated events that were reliably detected (JC ≥ 50) across all conditions were manually classified as upregulated, downregulated or unregulated by each knockdown, using the FDR and ΔPSI criteria (Supplementary File 3). RBPMS was classified by its responses in experiment 1. For each RBP knockdown, ΔPSI values were normalized to the ΔPSI of the PAC1 D versus P comparison in experiment 1. The resultant visualization (all event types: Supplementary Fig. S3; SEs only: Fig. 4A and B) shows RBP depletion induced changes that are concordant (blue, D → P) or discordant (red, P → D) with the dedifferentiation program, and reveals large sets of exons that are coordinately regulated by three (RBPMS, RBFOX2, MBNL1/2) or all four RBPs. Coregulation was particularly marked for Differentiation-included exons (D-exons, Fig. 4A). Just over half of the D-exons (83 of 158) were activated by RBPMS, and of these 89% (73/82) were co-regulated by at least one other RBP. Half of the RBPMS-activated exons (42/83) were co-activated by RBFOX2 and MBNL1/2, of which 27 were additionally activated by QK. For example, cassette exons in *Fat1* and *Mical3* were included in differentiated cells dependent upon activation by all four RBPs (Fig. 4C). All four RBPs regulated more than half of the D-exons, with the vast majority of effects concordant, rather than antagonistic, with the differentiation program (RBPMS 99%, RBFOX2 93%, MBNL 86%, QK 74%). Collaborative activity was also evident for exons more skipped in differentiated cells, but each RBP regulated a smaller proportion of these exons (Fig. 4B). While all four RBPs largely regulated SEs concordantly with the differentiation program, the percentage of the SE program antiregulated by RBPs increased in the order RBPMS (1.4%), RBFOX2 (9%), MBNL1/2 (13%), QK (19%). Notable examples where RBPMS, RBFOX2, and MBNL1 coregulated, but QK was antagonistic include the MXE pairs in *Actn1* (Fig. 4C) and *Tpm1*. The much tighter concordant regulation by RBPMS compared with the coregulators is consistent with it being the master regulator whose activity changes are responsible for switching a large proportion of the program.

The lower state of differentiation in experiment 2 means that we likely underestimated the contributions of RBFOX2 and QK to the differentiation program. For example, exon 22 of *Tns1* had a PSI of 19% in experiment 1, which reduced to 2%–3% upon knockdown of RBPMS or MBNL1 and in P-cells. However, in experiment 2 its basal level of inclusion was only 3%, so any further reductions would have missed our ΔPSI threshold. Likewise, *Smtn* exon 20, which is skipped under the influence of RBPMS and MBNL1/2 in D cells, had a PSI of 98% in experiment 2. Both exons were therefore classified as unregulated by RBFOX2 and QK although it remains possible that in more differentiated cells their regulatory role would have been evident. As an independent confirmation of RBP collaboration and to test whether we had overlooked some RBP contributions we used proliferative PAC1 cells in which RBPMS expression could be induced with doxycycline via the pInducer lentiviral system [38]. The resultant high levels of induced RBPMS can promote ASEs that are advanced differentiation markers [7, 15]. MBNL1/2, RBFOX2, and QK were knocked down both in basal conditions and after doxycycline induction of RBPMS (Supplementary Fig. S4A) and splicing patterns were assessed by RT-PCR (Supplementary Fig. S4B). *Tns1* exon 22 was confirmed to be activated by RBPMS (lane 5 versus 1), MBNL1/2 and RBFOX2, (lanes 7 and 8 versus 5), but unregulated by QK (lane 6 versus 5). *Smtn* exon 20 is more included in P cells and from RNA-Seq was classified as repressed by RBPMS and MBNL1/2, but unregulated by RBFOX2 and QK. In the pInducer cells all four RBPs were found to act as repressors of *Smtn* exon 20. The hCALD1 isoform, resulting from exon 3a and 4 inclusion, is usually only observed in tissue SMCs [7, 15] and was not detected in either of our RNA-Seq data-sets. In the pInducer PAC1 cells the *Cald1* event was found to be activated by all four RBPs, evident from increased inclusion upon RBPMS induction (lane 5 versus 1) and decreased inclusion upon RBP knockdown (lanes 6–8 versus 5). *Myocd* exon 2a, which had been detected in RNA-Seq experiments showed the expected responses (Supplementary Fig. S4B), being activated by RBPMS, MBNL1, and RBFOX2 but repressed by QK. These data therefore validate the findings from the RNA-Seq experiments but highlight that the full contributions of all RBPs, but especially RBFOX2 and QK are likely underestimated.

### Association of RBP binding motifs with regulated exons

**Figure 5.**
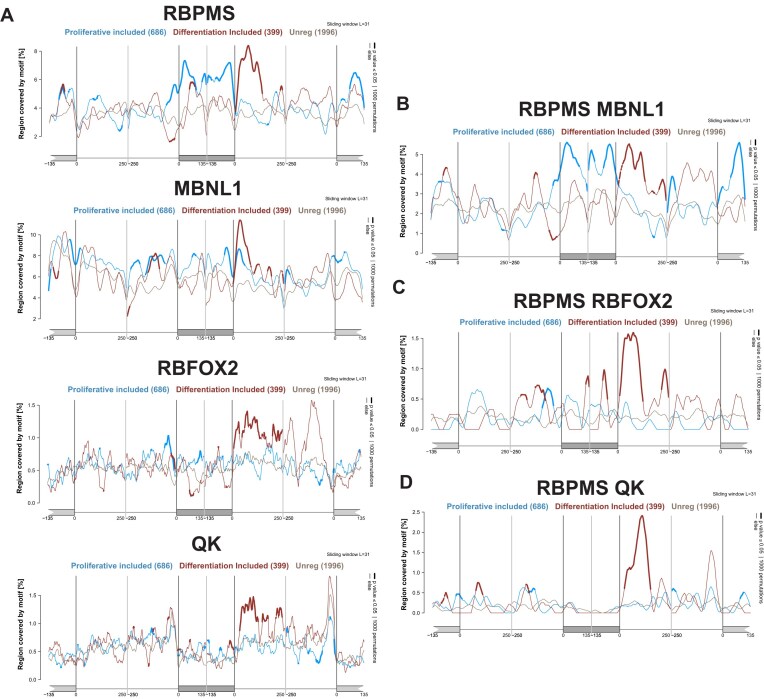
Splicing maps of RBP motifs associated with PAC1 differentiation regulated SEs. **(A)** RNA maps showing individual motif enrichment for RBPMS, MBNL1, RBFOX2, and QK across regulated SE events during PAC1 cell dedifferentiation. Red trace: exons more included in D-cells (*n* = 399); Blue trace: exons more included in proliferative cells (*n* = 686) (JC ≥ 50, |ΔPSI| ≥ 5%, FDR < 0.05). A background set of unregulated exons (*n* = 1996; JC < 50, |ΔPSI| < 5%, FDR > 0.1) was randomly downsampled from the total pool (grey trace). Motif enrichment was calculated using the MATT toolkit with a 31-nt sliding window. The *x*-axis spans ± 250 nt of the flanking introns and ± 135 nt of the cassette exon and adjacent constitutive exons. The *y*-axis indicates the percentage of nucleotides within each window covered by the motif. **(B–D)** RNA maps showing enrichment of linked motifs, in which the RBPMS motif (CACN₃₋₁₅CAC) is located within 5–25 nucleotides of the corresponding co-regulatory motif. **(B)** RBPMS-MBNL (linked motif: CACN₃₋₁₅CACN₅₋₂₅YGCY), **(C)** RBPMS-RBFOX2 (linked motif: CACN₃₋₁₅CACN₅₋₂₅GCATG), and **(D)** RBPMS-QK (linked motif: CACN₃₋₁₅CACN₅₋₂₅ACTAA/CTAAC). Enrichment patterns reflect spatial co-occurrence of RBPMS and partner RBP motifs on the same transcripts, consistent with cooperative regulation of exon inclusion. Statistically significant motif occurrences were determined by permutation testing (*P* ≤.05; 1000 iterations).

RBPs can regulate AS events by directly binding to transcripts in the vicinity of regulated exons, as part of larger complexes of regulators in which they do not themselves directly contact RNA [39], or indirectly by affecting the expression or activity of another regulatory RBP. In the absence of data on PAC1 cell transcriptome wide RNA binding by each of the RBPs, we looked for enrichment of RBP binding motifs associated with SEs regulated as part of the SMC program. Direct AS regulators, including RBPMS, RBFOX2, MBNL1/2 and QK, frequently exhibit position-dependent activity, with binding upstream or within exons associated with repression of splicing and binding downstream of the exon associated with activation [40]. To validate motif-terms for each RBP, we first generated RNA Maps using the sets of exons that had been regulated by knockdown of the cognate RBP. Each RBP knockdown splicing map showed clear downstream peaks associated with activation (Supplementary Fig. S5A, red traces), and upstream or exonic peaks associated with splicing repression (blue traces). These peaks disappeared upon point mutations to the motifs (Supplementary Fig. S5B). Having validated the RNA motifs associated with each RBP we next generated splicing maps using the SEs regulated between PAC1 D and P cells (Fig. 5A). The PAC1 RBPMS map showed significant downstream activation and upstream/exonic repression peaks (Fig. 5A), very similar to the RBPMS KD map, supporting direct action of RBPMS on the PAC1 program. RBFOX, MBNL, and QK maps also showed strong downstream activation peaks; however, they did not feature the prominent repression peaks seen in their cognate knockdown maps (compare Fig. 5A with Supplementary Fig. S5A). This supports a direct role for all four RBPs, at least in the activation of differentiation exons. To address whether the different enriched motifs occurred on the same transcripts, we generated RNA maps using linked RBPMS-RBFOX, RBPMS-MBNL, and RBPMS-QK motifs separated by up to 25 nt (Fig. 5B–D, respectively). We again observed prominent downstream activation peaks for each motif combination, but also upstream and exonic repression peaks (Fig. 5B and Supplementary Fig. S5C). The RNA Maps of individual and combined RBP motifs suggest that their collaborative activity, particularly evident in the activation of differentiation exons (Fig. 4A), is likely mediated by direct binding of combinations of RBPs around regulated exons.

### IDR mediated interactions are essential for coregulation

To further investigate the basis of coregulation by the four RBPs we carried out co-IP experiments to identify physical interactions, followed by knockdowns combined with complementation by overexpressed proteins. We aimed to identify mutations that impaired protein-protein interactions, and to ask how these mutations affected splicing activity. PAC1 cells are not suitable for these experiments as the efficiency of plasmid transfection is low, meaning that only splicing of co-transfected minigene constructs can be interrogated. We therefore used HEK293T cells, in which RBPMS expression is sufficient to switch splicing of SMC-specific ASEs in endogenous transcripts [7, 41]. Consistent with this the measured concentration of RBPMS (31 nM) in HEK293T cells is ∼20-fold lower than RBFOX2 (590 nM), MBNL1 (650 nM), and QK (710 nM) [42].

**Figure 6.**
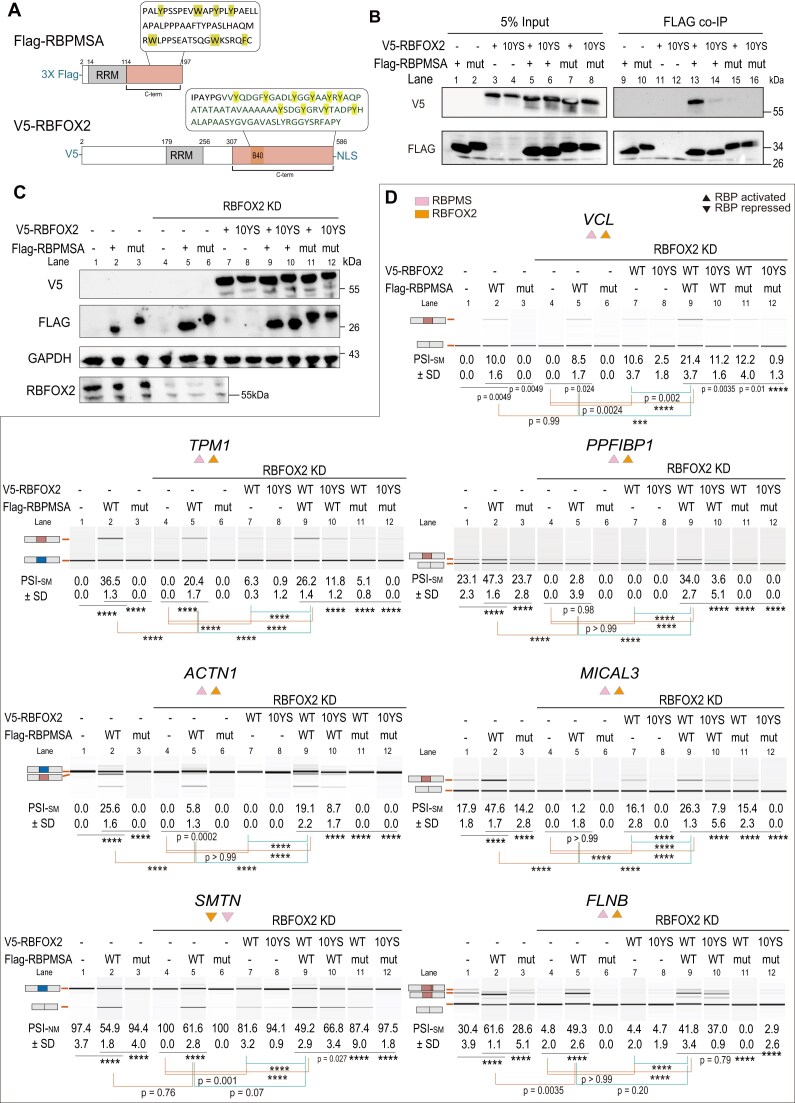
Aromatic residues in RBPMS and RBFOX2 are essential for physical and functional interaction. **(A)** Schematic representation of RBPMSA and RBFOX2 constructs used in the co-IP assays, showing the RNA recognition motif, C-terminal regions, and positions of aromatic mutations. **(B)** Co-IP analysis showing that mutation of aromatic residues within RBPMSA or the Y-region of RBFOX2 disrupts their physical association. HEK293T cells were co-transfected with FLAG-tagged RBPMSA (wild-type, WT; or aromatic mutant, mut) and V5-tagged RBFOX2 (WT or 10YS mutant). FLAG immunoprecipitates were probed with anti-V5 and anti-FLAG antibodies. Inputs represent 5% of total lysate. **(C)** Western blot controls for the RT-PCR rescue experiments inpanel(D), verifying RBFOX2 knockdown and expression of V5-RBFOX2 (WT or 10YS) and FLAG-RBPMSA (WT or mut). GAPDH serves as a loading control. Protein molecular weights (kDa) in panels (B) and (C) are indicated. **(D)** RT-PCR analysis of SM-associated splicing events in RBFOX2 knockdown HEK293T cells, co-expressing combinations of RBPMSA (WT or mut) and RBFOX2 (WT or 10YS). Representative events include *TPM1, VCL, ACTN1, MICAL3, PPFIBP1, SMTN*, and *FLNB*. Lanes 1–3 represent control siRNA (no KD) with RBPMSA−/WT/mut; lanes 4–6 represent RBFOX2 knockdown with RBPMSA−/WT/mut; lanes 7–12 represent RBFOX2 knockdown with co-expression of RBFOX2 WT or 10YS in combination with RBPMSA WT or mut. Statistical significance was determined by two-way ANOVA with multiple comparisons; ns and *, **, *** *P*-values are indicated on the graphs, and **** denotes *P* <.0001. Error bars represent mean ± SD. Data shown are from one representative experiment performed in triplicate. Coloured triangles above each event indicate the inferred activity of each RBP, namely activation (▲), repression (▼), or no significant effect (●), as determined from their behaviour in PAC1 knockdown experiments.

To test whether RBPMS interacts with each of the other RBPs we expressed pairs of epitope-tagged proteins (FLAG-RBPMS, Venus-MBNL1, V5-RBFOX2, Myc-QK; Fig. 5E) in HEK293T cells and carried out anti-FLAG immunoprecipitations after benzonase treatment to abolish RNA-bridged interactions. RBPMS coimmunoprecipitated each of the other tagged RBPs (Fig. 6A and B, and Supplementary Figs S6A and B, and S9A and B). Moreover, each interaction was disrupted by mutation of seven C-terminal aromatic residues (“mutRBPMS”, Fig. 6A and B, and Supplementary Figs S6B and S9B) that are important for RBPMS activity (Fig. 6A and D, and Supplementary Figs S7B and S10B).

To further investigate the relationship between physical and functional interactions we looked for mutants of RBFOX2, MBNL1 and QK that are impaired for interaction with RBPMS. RBFOX1 higher order oligomerization is mediated by a cluster of tyrosines within its C-terminal IDR; mutation of ten tyrosine residues to serine (10YS mutant) led to loss of self-association and activity [43]. The equivalent 10YS mutant of rat RBFOX2 (Fig. 6A) failed to interact with RBPMS (Fig. 6B, lanes 13 and 14) and also lost splicing activity (Fig. 6D, lanes 7 versus 8 and 9 versus 10). The specificity of the 10YS effect upon interaction with RBPMS was demonstrated by the continued interaction of the mutant with MBNL (Supplementary Fig. S8). In contrast, we were unable to identify specific regions of MBNL1 and QK responsible for interaction with RBPMS. All members of a set of N- and C-terminal MBNL1 deletion mutants coimmunoprecipitated with RBPMS (Supplementary Fig. S6C and D), suggesting redundant interactions between different parts of MBNL1 and RBPMS. We identified a cluster of four tyrosines in QK5 as a potential RBPMS interacting region (Supplementary Fig. S9A); however, deletion of this region (285–293) had no effect upon interaction with RBPMS (Supplementary Fig. S9C). In summary, the co-IP data show that RBPMS interacts with each of the other RBPs via aromatic residues in its C-terminal IDR, and that the RBFOX2 C-terminal tyrosine cluster is reciprocally needed for interaction with RBPMS.

To investigate functional collaboration we overexpressed RBPMS (WT or aromatic mutant) in HEK293T cells in combination with knockdown and complementation of each of the other RBPs, and tested the effects upon seven RBPMS regulated ASEs (Fig. 6D). Overexpression of RBPMS regulated all seven ASEs (Fig. 6D, lanes 1 and 2) and in every case the RBPMS aromatic mutant was inactive (Fig. 6D lanes 2 and 3). All events responded to RBFOX2 knockdown either under conditions of RBPMS overexpression (lanes 2 versus 5), or for some events under baseline conditions (lanes 1 versus 4); in all cases RBPMS and RBFOX2 coregulated in the same direction. Overexpression of WT RBFOX2 partially or fully complemented RBFOX2 knockdown (lanes 7 versus 4 and/or 9 versus 5) for all but one event (*FLNB*). By contrast, the RBFOX2 10YS mutant was inactive (lanes 7 versus 8, 9 versus 10). Thus, mutations of IDR aromatic residues in both RBPMS and RBFOX2 lead to reciprocal loss both of activity and their mutual interaction. Of the six events that responded to RBFOX2 overexpression, three showed additive responses to RBPMS and RBFOX2 (*TPM1, VCL, SMTN*), while three showed synergistic responses (*PPFIBP1, MICAL3, ACTN1* SM exon inclusion). Strikingly, PPFIBP1 exon inclusion had a negligible response to RBPMS or RBFOX2 individually (PSI below 3%), but was included to > 30% when both WT proteins were expressed. Aromatic mutations in either protein reduced exon inclusion to basal levels (Fig. 6D, *PPFIBP1*, lanes 9–12).

Five of the events responded as expected to MBNL1 knockdown, with four responding additively (*TPM1, ACTN1, FLNB*) or synergistically (*VCL*) to RBPMS and MBNL1 overexpression (Supplementary Fig. S7, lanes 4, 5, 7, and 8). In contrast, PPFIBP1 and *MICAL3* showed unexpected antagonistic responses to MBNL1 and RBPMS overexpression. With QK, six events responded to knockdown as expected (Supplementary Fig. S10). PPFIBP1 was classified as QK unregulated in PAC1 cells (Fig. 4) and was also unaffected in HEK293T cells (Supplementary Fig. S10). *FLNB* alone was nonresponsive to QK knockdown, despite being classified as QK activated in PAC1 cells. Four of the events responded as expected to RBPMS and QK5 overexpression; TPM1, *ACTN1*, and *VCL* were antagonistically regulated, while *SMTN* was coregulated (Supplementary Fig. S10). The failure of some events to respond to overexpression of RBFOX2, MBNL1, and QK, despite in some cases responding to knockdown, could be related to the use of a single overexpressed isoform of each protein, whereas each protein has multiple isoforms with variable activities.

Taken together, these data indicate that RBPMS can coregulate SMC-specific ASEs when expressed exogenously in HEK293T cells and that this activity correlates with its ability to interact with the other RBPs mediated by C-terminal aromatic residues. In the case of RBFOX2, this is mirrored by the dependency on C-terminal tyrosines both for interaction with RBPMS and for coregulation of splicing (Fig. 6).

### RBPMS promotes contractile phenotype properties

**Figure 7.**
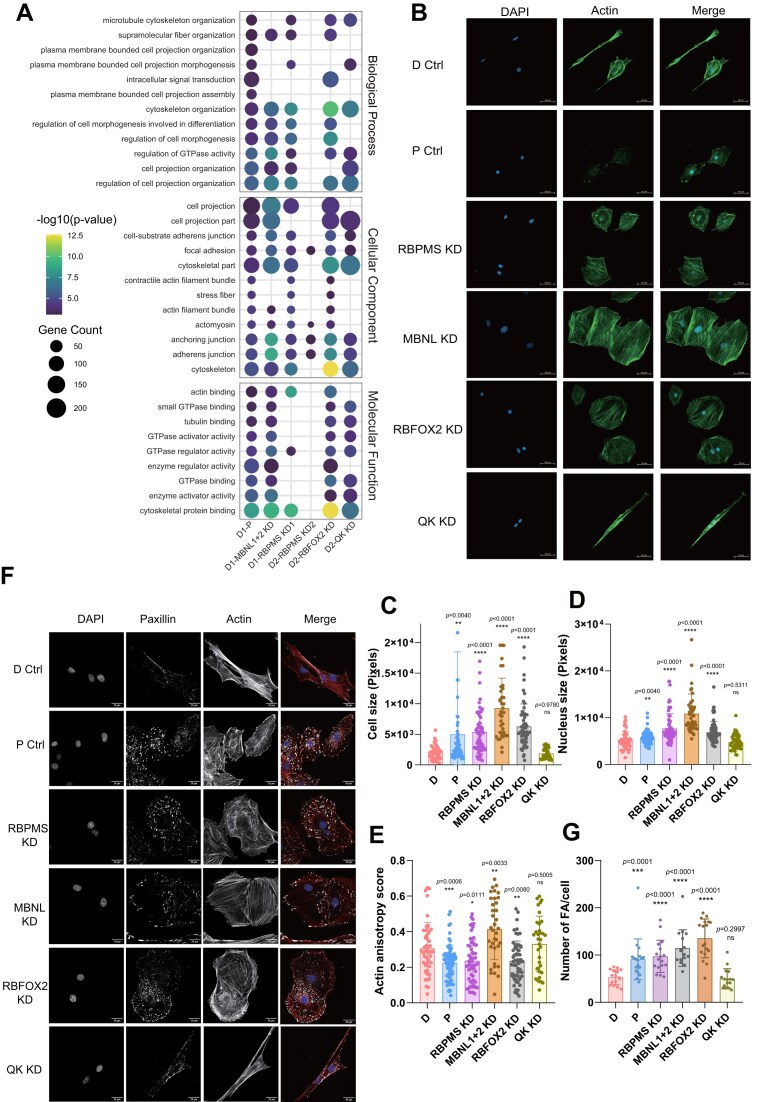
Cytoskeletal and adhesion-related features regulated by RBPMS, MBNL1, RBFOX2, and QK in PAC1 D cells. **(A)** GO enrichment analysis of genes significantly affected by AS (all event types) in PAC1 cells during dedifferentiation and following RBP knockdown in differentiated cells. Selected smooth muscle-related GO terms were derived from the D1 versus P comparison. Dot size indicates the number of associated genes, and colour represents the −log_10_(*P*-value) of enrichment. Terms are grouped by GO category: Biological Process, Cellular Component, and Molecular Function. **(B)** Immunofluorescence staining of PAC1 cells under the indicated conditions. DAPI stains nuclei (blue), and phalloidin stains F-actin (green) to visualize cytoskeletal structures. Images were acquired using a fluorescence microscope. Rows correspond to D Ctrl, P Ctrl, and knockdowns of RBPMS, MBNL1 + 2, RBFOX2, and QK in differentiated PAC1 cells. Scale bars, 50 μm. **(C–E)** Quantitative analysis of cell morphology from panel (B). **(C)** Cell area, **(D)** nuclear area, and **(E)** actin anisotropy scores were quantitated using ImageJ. Actin anisotropy was specifically quantified using the FibrilTool macro. Sample sizes were as follows: **(C)** number of cells analysed for cell area, *n* = 44, 68, 60, 37, 59, 34; **(D)** number of nuclei measured, *n* = 47, 74, 41, 71, 49; **(E)** number of cells analysed for actin anisotropy, *n* = 47, 68, 60, 37, 49. Statistical significance was determined using the Wilcoxon Mann–Whitney test (*P* <.05, *P <.01, **P <.001, ***P <.0001). **(F)** High-resolution immunofluorescence images of PAC1 cells stained with DAPI (nuclei), paxillin (FA), and phalloidin (F-actin), acquired using a confocal microscope. Merged images show overlay of all three channels. Scale bars, 20 μm. **(G)** Quantification of FA number per cell based on paxillin staining in panel **(F)**. Statistical significance was assessed using the Wilcoxon Mann–Whitney test (*P* <.05, *P <.01, **P <.001, ***P <.0001). Sample size for each group is *n* = 16, 18, 17, 16, 18, 16, representing the number of cells analysed. Data are from one representative biological replicate; similar trends were observed across three independent experiments.

**Figure 8.**
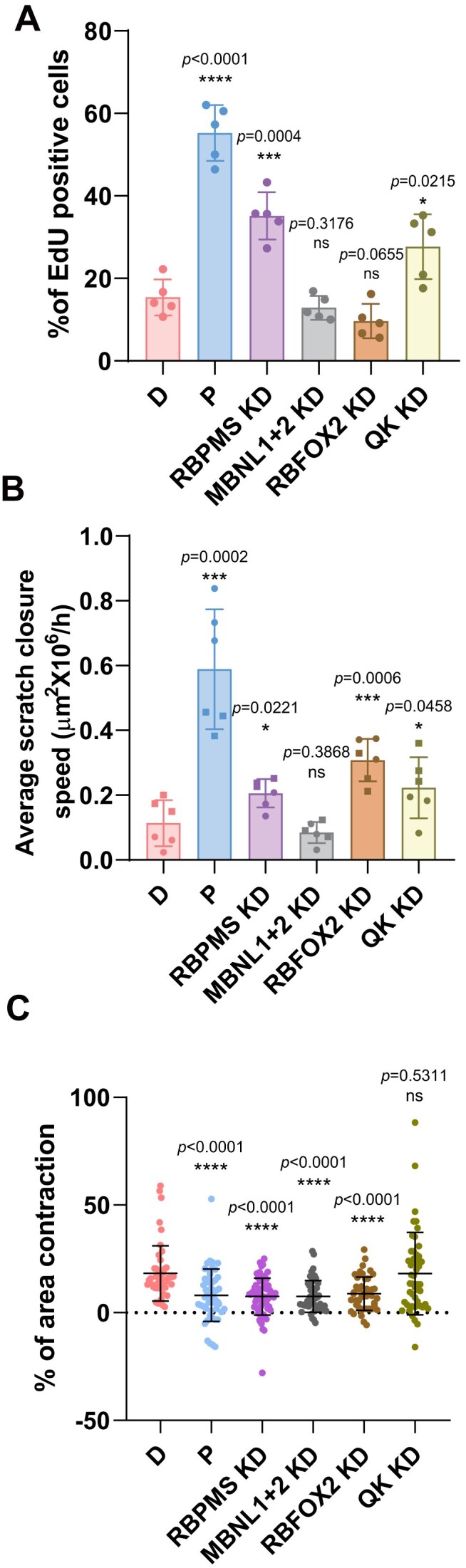
RBPMS and its cofactors regulate PAC1 D cell proliferation, migration, and contractility. **(A)** EdU incorporation assay measuring DNA synthesis in PAC1 cells under the indicated conditions. Data are presented as the percentage of EdU-positive nuclei per total cells (*n* = 5 replicates per group). Results shown are from one representative experiment performed in biological triplicate. **(B)** Wound healing assay measuring the average scratch closure speed (μm² × 10^6^/h) in PAC1 monolayers. Closure speed was calculated from the slope of the linear regression line, corresponding to the rate of gap closure. Quantification was performed over 8 h to minimize the influence of cell proliferation. The graph represents two independent biological experiments, distinguished by different symbol shapes. Statistical significance was determined using unpaired *t*-tests (*P <.05, **P <.01, ***P <.001, ****P <.0001). **(C)** Contraction assay measuring the percentage of area contraction in PAC1 cells cultured on glass substrates. Changes in cell area were quantified following a 15-min treatment with the Ca²⁺ ionophore ionomycin. Each data point represents an individual cell (*n* = 48, 49, 70, 45, 48, 47). Results shown are from one representative experiment performed in biological triplicate. Statistical significance was determined using the Wilcoxon Mann–Whitney test (*P* <.05, *P <.01, **P <.001, ***P <.0001; ns, not significant). Error bars indicate mean ± SD. All RBP knockdowns were performed in differentiated PAC1 cells.

To understand the impact of RBP-regulated AS on SMC function, we first examined the enriched categories of genes containing significantly regulated ASEs. GO analysis revealed that RBPMS, RBFOX2, MBNL1/2, and QK all preferentially target genes involved in cytoskeletal organization, actin filament binding, FA, and cell projection assembly—macromolecular assemblies that are central to SMC morphology, motility and contractility (Fig. 7A and Supplementary File 4). Notably, these GO terms were also significantly enriched among ASEs regulated during PAC1 dedifferentiation, suggesting that RBP-mediated splicing program plays a key role in driving SMC phenotypic transitions. To directly test the contributions of the RBPs to differentiated PAC1 cell phenotype we measured a variety of morphological features (cell size, nuclear size, actin anisotropy, FA number, Fig. 7) and cell behaviours (proliferation, motility and contraction; Fig. 8 and Supplementary Fig. S11) in PAC1 D and P cells and in PAC1 D cells after RBP knockdown. Western blots and RT-PCR of candidate ASEs confirmed effective RBP knockdown in PAC1 D cells (Supplementary Fig. S11A and B).

Compared to the spindle shape of D cells, PAC1 P cells have a rounder and flatter shape with a larger cell and nuclear area (Fig. 7B–D). They also have lower amounts of F-actin, with filaments showing less parallel ordering (lower actin anisotropy, Fig. 7B and E). Depletion of RBPMS or RBFOX2 phenocopied the D to P transition in each of these respects (Fig. 7B–E), with the exception of reduced actin levels, which likely result from transcriptional downregulation in PAC1 P cells (Fig. 2C; ACTA2 is 80-fold downregulated). MBNL depletion produced the largest increases in cell and nuclear area but, contrasting with dedifferentiation, this was accompanied by increased (∼1.3-fold) actin anisotropy. QK depletion had no effect on any of these features; many cells fully maintained the spindle appearance of control differentiated cells (Fig. 7B, bottom row), although there was some heterogeneity in cell shape. FA, mechanosensitive structures that connect the actin cytoskeleton with attachments to the extracellular matrix and are important for both motility and contraction, were visualized by immunofluorescence for the FA marker paxillin (Fig. 7F). The number of FAs was higher in PAC1-P compared to D cells, and knockdown of RBPMS, RBFOX2, and MBNL1/2, but not QK, also increased their number (Fig. 7F and G). In summary, only RBPMS and RBFOX2 depletion phenocopied dedifferentiation by all morphological criteria, while QK had no effect.

We next measured three functional properties—proliferation, motility, and contraction—that differ between SMC phenotypes. Cell proliferation, measured by EdU incorporation, was 3.6-fold higher in P versus D cells, as expected (Fig. 8A and Supplementary Fig. S11C). Knockdown of RBPMS and QK in PAC1 D cells also increased proliferation by 2.3- and 1.8-fold, respectively, while depletion of RBFOX2 and MBNL1/2 had no effect. Cell motility was assessed by a “scratch closure” assay over 8 h. PAC1-P cells showed 5.2-fold higher motility than D cells, and knockdown of RBPMS, RBFOX2, and QK in PAC1 D cells increased motility 1.8-, 2.7-, and 2.0-fold respectively, while MBNL depletion had no effect (Fig. 8B and Supplementary Fig. S11D). Finally, we tested whether RBPs influence contraction—a hallmark feature of differentiated cells. Individual cells were imaged on glass substrates and changes in cell area quantified following a 15-min treatment with the Ca^2+^ ionophore ionomycin. This assay bypasses signalling pathways that lead to increases in cytoplasmic Ca^2+^, thereby directly assaying intrinsic differences in cellular contractile machinery. As expected, PAC1-P cells were significantly less contractile than D cells (Fig. 8C and Supplementary Fig. S11E). Knockdown of RBPMS, RBFOX2, and MBNL1/2 in PAC1 D cells all reduced contractile activity to similar levels as PAC1-P cells. In contrast, QK knockdown had no effect on contraction.

In summary, knockdown of the four RBPs had various effects on PAC1 cell morphology and function, consistent with their widespread transcriptomic effects focused on the actin cytoskeleton and cell adhesion machineries. Remarkably, RBPMS stood out as the only RBP whose knockdown consistently caused changes towards a less differentiated, more proliferative phenotype in all morphological and functional assays (Figs 7 and 8).

## Discussion

### An RBPMS led splicing regulatory axis

Our data show that the SMC master splicing regulator RBPMS works in coordination with three more widely expressed RBPs—RBFOX2, MBNL1, and QK—to drive a cell-state specific AS program that promotes differentiated SMC morphology and contractile behaviour, with low motility and proliferation. RBPMS depletion also led to statistically significant reduction in expression of SMC differentiation markers. However, the magnitude of changes was a small fraction of the decreases seen in proliferative PAC1 cells; for example, *Myocd* was reduced 1.7-fold by RBPMS knockdown but 30-fold in proliferative cells. In stark contrast, for 25% of all ASEs regulated by dedifferentiation including those with the highest ΔPSIs, RBPMS knockdown produced ΔPSIs of comparable magnitude to dedifferentiation (Fig. 2F).

Of the four RBPs tested, RBPMS affected the fewest ASEs, but importantly it showed almost complete concordance with PAC1 differentiation (Fig. 4). Depletion of each of the other three RBPs discordantly affected a higher proportion of the differentiation AS program as well as dysregulating numerous ASEs that are not part of the program. Notably, the magnitude of ΔPSI responses induced by MBNL, RBFOX, and QK knockdown showed a higher range than RBPMS knockdown (Figs 2D and 3D), and many of the highest ΔPSI changes were in events unregulated during PAC1 differentiation. These wider effects reflect the even expression of these RBPs in both differentiated and proliferative VSMC states (Fig. 1B and C), helping to maintain the balance of many ASEs that do not change during this transition, as well as collaborating with RBPMS in the regulated changes and helping to set the basal level of inclusion of some of these events in P-cells. Notably, PAC1-D cells are only partially differentiated with lower levels of RBPMS and a less advanced splicing program than is observed in VSMCs *in vivo* [6, 7, 15]. We anticipate that in fully differentiated VSMCs *in vivo* both the number and magnitude of splicing changes induced by RBPMS depletion would be higher.

Cooperation between RBPMS, RBFOX2, and MBNL1/2 was anticipated [10, 15, 19]. In contrast, QK was expected to promote a less differentiated more proliferative phenotype based partly on its known antagonistic activity on a small number of RBPMS regulated events [6, 7]. However, QK has been reported to promote both proliferation [9] and differentiation [44] of VSMCs suggesting that it has context-specific activity that is modulated by the activities of other RBPs. Indeed, both QK and RBFOX2 were shown to repress inclusion of the *Flnb* H1 exon in breast cancer cells [20] but in PAC1 cells all four RBPs are activators of the *Flnb* exon (Fig. 4).

The predominantly concordant role of the coregulators with the PAC1 differentiation AS program, and the co-enrichment of known binding motifs for the RBPs with SMC regulated exons (Fig. 5 and Supplementary Fig. S5) could reflect completely independent action of each of the four RBPs with common target RNAs. However, a number of observations argue for more direct collaborative action. First, we observed co-IP of RBPMS with all three coregulators and of MBNL1 with RBFOX2 (Fig. 6B, and Supplementary Figs S6B and S9B). This agrees with RBPMS BioID interaction data from neonatal mouse cardiomyocytes identified RBFOX1, MBNL1, and QK [16]. All interactions with RBPMS were disrupted by C-terminal aromatic residue mutations that also abolished RBPMS splicing activity (Fig. 6, and Supplementary Figs 6 and 9). The interactions were resistant to benzonase, so are not mediated by RNA, but could be bridged by other proteins. The reciprocal effects of aromatic mutations in RBFOX2 (10YS mutant) upon both splicing activity and interaction with RBPMS (but not MBNL1) are consistent with, but do not prove, a direct interaction between IDRs with complementary molecular grammar [45]. The nature of any such direct interactions could be probed directly *in vitro* with a range of biophysical approaches. RBPMS is ideal for such approaches as full length recombinant protein can be assayed for both activity and biophysical properties in cell free systems [19]. Second, using HEK293T cells, we could show that for many target ASEs not only were RBPMS and the coregulators nonredundant, with additive effects, but in some cases we observed synergistic responses indicating strong co-dependence of pairs of factors. This was particularly evident with RBFOX2 and RBPMS action upon PPFIBP1 where only the combination of both WT proteins was able to restore exon inclusion (Fig. 6D). Likewise, synergy between RBPMS and MBNL1 was observed upon meta-VCL exon inclusion (Supplementary Fig. S7). For some events (e.g. FLNB with RBFOX2) we did not observe complementation of knockdown by overexpression. RBFOX2, MBNL1, and QK all have multiple isoforms, so it is plausible that other isoforms expressed in HEK293T cells might also be required for function upon some target events.

Mechanisms by which the RBPs coregulate could involve cooperative binding to RNA, possibly as a preformed complex similar to the RBFOX associated LASR complex [39, 43]. Alternatively, regulatory activity could arise only upon binding of the required combination of RBPs, with their distinct effector domains. These possibilities could be distinguished via transcriptome-wide RBP–RNA binding assays, such as CLIP [46] or ARTR-Seq [47], or via single molecule approaches *in vitro* [48]. Notably, RBFOX2, QK, and MBNL1 have been shown to coactivate a striated-muscle specific *Snap23* microexon [49], so the RBPMS regulatory axis in SMCs may be a variant on a more widespread regulatory strategy. It would also be interesting to investigate how the phosphorylation of RBPMS by ERK, which acutely downregulates RBPMS activity [41], affects interactions between the coregulators and their binding to RNA.

### PAC1 cells model phenotypic plasticity

PAC1 cells were originally reported to express molecular markers of differentiated VSMCs and to respond to vasoactive agonists with elevated cytoplasmic Ca^2+^ [21]. We subsequently used PAC1-D and P cells to model the transcriptional and AS splicing changes of VSMC phenotypic plasticity with accompanying changes in cell shape and actin organization [6, 7]. Here, for the first time, we show that PAC1-P and PAC1-D cells also exhibit reciprocal functional differences in proliferation, migration, and contraction (Fig. 8), as well as changes in FA organization (Fig. 7) [7]. The full or partial phenocopying of all these features upon RBPMS knockdown, in the absence of the transcriptional program, attests to the important role of the AS program in phenotypic plasticity. Of interest, the RBP knockdowns showed uncoupling of some of the concerted changes that occur in phenotypic plasticity. For example, upon QK knockdown, PAC1-D cells maintained their spindle shape and contractile activity, yet gained proliferation and motility activities (Figs 7 and 8).

Nevertheless, the PAC1 cell model has limitations. In particular, the degree of differentiation, which never approaches that of mature VSMCs *in vivo*, varies between experiments, as highlighted by the lower degree of differentiation of PAC1-D cells in the RBPMS/RBFOX2/QK knockdown experiment (Fig. 3 and Supplementary Fig. S2C and D). While we were able to identify numerous RBFOX2 and QK regulated ASEs, we were unable to assess their potential activation of exons whose basal inclusion levels were too low in experiment 2 (e.g. *Tns1* exon 22). This means that the degree of coregulation of RBFOX2 and QK with RBPMS is more extensive than we observed. Notably, the converse is not true; antagonism by RBFOX2 or QK of differentiated AS events (e.g. inhibition of *Tns1* exon 22) would have been evident. Nevertheless, our data provide the first insights into the clearcut role of an RBPMS led axis of RBPs in controlling SMC plasticity.

### Contribution of the RBPMS-axis to differentiated phenotype

RBPMS-regulated ASEs, like the PAC1 D-P program, are highly enriched for proteins associated with actin filaments and FA. Indeed, proteins such as ACTN1, FLNB, and FAT1 (Fig. 4) are involved in both processes. The actin cytoskeleton and FA are critical to the function of both differentiated cells, characterized by actomyosin mediated contraction, and proliferative cells in which actin drives motility. The RBPMS-driven AS program appears to remodel these machineries for their different functions. The degree to which RBP knockdowns phenocopied dedifferentiation varied by function, with full loss of contraction to the level of P-cells (RBPMS, MBNL1, RBFOX; Fig. 8C), but only partial increases in motility (RBPMS, RBFOX2, QK; Fig. 8B) and proliferation (RBPMS, QK; Fig. 8A). These different responses likely reflect the necessity but insufficiency of the splicing program for cell phenotypes. So loss of the essential D-cell AS program renders the cells noncontractile, but without the accompanying transcriptional program, involving downregulation of contractile actin and myosin genes, AS changes are not sufficient for full acquisition of motility.

The variable responses of different cellular properties (Figs 7 and 8) to RBP knockdowns suggests the possibility to identify cognate groups of ASEs (Fig. 4) that responsible for individual phenotypes. However, there are important caveats to this approach. First, MBNL1, RBFOX2 and QK knockdown all lead to numerous changes in AS and mRNA abundance that are unregulated during dedifferentiation, many in genes associated with cytoskeletal functions. For example, the striking increase in F-actin filament alignment only upon MBNL knockdown (Fig. 7), potentially due to events regulated by MBNL alone, might prevent the increases in motility caused by dysregulation of events co-regulated by all four RBPs. Second, as noted above, we have underestimated the degree of coregulation (but not antagonistic regulation) by RBFOX2 and QK. With these caveats in mind, ASEs associated with the full loss of contraction might be expected to be coregulated by RBPMS and MBNL1, and either unregulated or antagonistically regulated by QK. The 99 ASEs satisfying these criteria are in 71 genes enriched for functional terms associated with muscle development and structure. These include the transcriptional coactivator MYOCD, as well as numerous actin associated proteins including ACTN1, ACTN4, SVIL, PDLIM5, PDLIM7, SMTN, TPM1, and TPM2, as well as membrane Ca^2+^ pumps (ATP2A2, ATP2B4). The altered splicing events of actin-associated proteins are possible drivers of loss of contraction. Our contraction assay (Fig. 8) used a Ca^2+^ ionophore to allow direct assessment of contractile machinery function. The altered splicing of proteins involved in regulating cytoplasmic Ca^2+^ suggests that earlier steps in excitation-contraction might also be modulated by the RBPMS-axis.

Events associated with the partial gain in motility (Fig. 8B) are expected to be coregulated by RBPMS, RBFOX2, and QK. Using the less stringent criterion that events should not be antagonistically regulated by RBFOX2 or QK identifies 220 ASEs, many overlapping with the contractile candidates and with enrichment for actin filament based processes. Three of the nonoverlapping candidates with the largest ΔPSIs upon dedifferentiation and RBPMS knockdown are coactivated by all four RBPs and associated with actin: *Flnb, Fat1*, and *Mical3*. For the latter two, the ASEs are predicted to have important functional consequences that could impact motility. Inclusion of the 36 nt penultimate exon of the *Fat1* gene (Fig. 4C), which encodes an atypical cadherin, disrupts a phosphotyrosine binding motif in the FAT1 intracellular domain [50]. The longer isoform fails to localize to cell leading edges and specific knockdown of this isoform enhanced motility of NRK-52E cells, whereas knockdown of all isoforms inhibited motility [50]. Inclusion of this exon under the influence of RBPMS/MBNL/RBFOX and QK could therefore contribute to the lower migratory activity of differentiated cells (Fig. 8). MICAL3 is an NADPH mono-oxygenase with an N-terminal monooxygenase domain, Calponin Homology domain, LIM domain, and a C-terminal coiled coil domain [51]. The monooxygenase domain oxidizes Met 44 and 47 in F-actin to induce depolymerization. This activity is restrained via autoinhibitory interaction with the coiled coil region [52]. Conversely interaction of the coiled coil region with Rab-GTP proteins and plexin signalling effectors relieves the autoinhibition. The 17 aa insert resulting from the *Mical3* 51 nt exon inclusion (Fig. 4C) is immediately adjacent to the coiled coil domain, so has the potential to modulate the autoinhibitory activity. For example, enhancing the autoinhibition might help to stabilize F-actin filaments in nonmotile cells.

Proliferation was upregulated by RBPMS and QK depletion (Fig. 8A), consistent with other reports that RBPMS is antiproliferative and can act as a tumor suppressor [15–17, 53]. Genes affected by RBPMS and QK regulated ASEs showed no enrichment for functions associated with cell proliferation, and no obvious candidate events in individual genes. It is therefore possible that the proliferative response is a downstream consequence of the directly targeted processes.

Although our data point to a central role for RBPMS in regulation of differentiated VSMCs via a regulated splicing program, this does not preclude roles at other levels of gene expression, and in other tissues. Indeed, RBPMS and its paralog RBPMS2 also play important roles in cardiomyocytes [16, 54–57] and in priming ES cells for subsequent differentiation to cardiomyocytes via widespread translational regulation [58]. Mouse conditional knockout models have revealed contributions of RBPMS to both normal cardiomyocyte and VSMC function. Embryonic deletion of RBPMS leads to lethality by P4 with cardiac defects including premature terminal differentiation of cardiomyocytes [16], while knockout in adult cardiomyocytes leads to defects in contractility and dilated cardiomyopathy [56]. However, the patent ductus arteriosus in embryonic RBPMS knockout mice is likely related to hyperproliferation of VSMCs [16]. Likewise, *in vivo* VSMC-specific deletion or overexpression of RBPMS lead to dysregulated VSMC proliferative responses, consistent with RPMS promoting a contractile VSMC state [17]. These effects were principally interpreted via alterations in *Myocd* exon 2a splicing [17], which had previously been shown to be a direct target of both RBPMS and QK, which regulate it antagonistically [7, 9]. We now find that MBNL1 and RBFOX2 also co-activate *Myocd* exon 2a inclusion along with RBPMS. Given the very modest changes in transcription upon RBPMS depletion in PAC1 D-cells (Figs 2C and 3C) it is likely that the wider RBPMS regulated splicing program, with functional enrichment for genes encoding numerous proteins involved in the actin cytoskeleton and cell adhesion, has a larger contribution to the differentiated phenotype both in the PAC1 cell culture model and *in vivo*. Given the proposed central role of the RBPMS-driven splicing network in VSMCs it is likely that its dysregulation will be associated with pathology. The involvement of MBNL1 in the RBPMS-axis is intriguing as its dysregulation is associated with the pathology of myotonic dystrophy not only in skeletal and cardiac muscle [59] but also potentially in gastrointestinal visceral SMCs [60].

In summary, we show that RBPMS, in collaboration with MBNL1, RBFOX2, and QK, drives a set of splicing changes that contribute to the contractile SMC phenotype via a concerted set of regulated splicing events. Future work will aim to unravel the contributions of individual or groups of ASEs to the observed phenotypes.

## Supplementary Material

gkaf1386_Supplemental_Files

## Data Availability

Raw and processed RNA-seq data have been deposited in NCBI GEO under accession numbers GSE301925 and GSE210586. The analysis and dataset are publicly available through the bulkAnalyseR interface at https://mohorianulab.org/shiny/CSmith/Huang2025/.
