# Supplementary material for: An RBPMS-driven splicing regulatory axis including MBNL1, RBFOX2, and QK promotes smooth muscle cell contractile identity
Source: Nucleic Acids Res. 2025 Dec 22;53(22):gkaf1386. doi: 10.1093/nar/gkaf1386 (PMC12721327; doi:10.1093/nar/gkaf1386)
Supplement: gkaf1386_Supplemental_Files [file gkaf1386_supplemental_files.zip › Huang et al Supplementary Figures.pdf]

Supplementary Figures S1-S11

An RBPMS driven splicing regulatory axis including MBNL1, RBFOX2 and QK promotes smooth muscle cell contractile identity

Yuling Huang, Rafael Kollyfas, Ruth Partridge, Clare Gooding, Sanjay Sinha, Irina Mohorianu, Aishwarya Jacob, & Christopher W.J. Smith

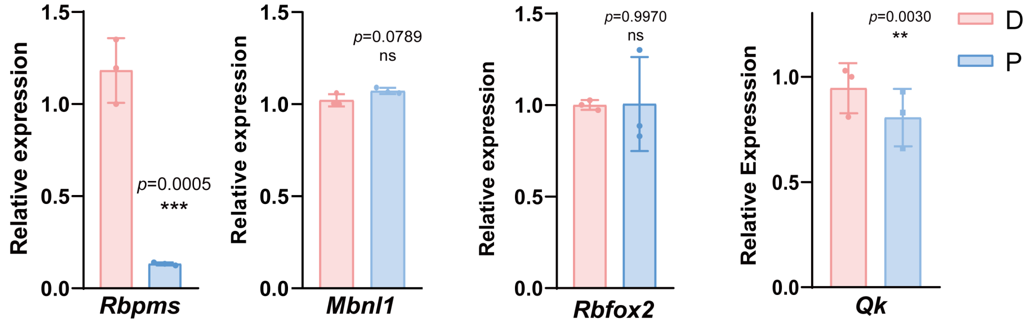

Supplementary Figure S1. RT-qPCR verification of *Rbpms*, *Mbnl1*, *Rbfox2* and *Qk* mRNA relative expression between PAC1 D and P cells. The relative expression levels were normalised to *CanX* and *Gapdh*.

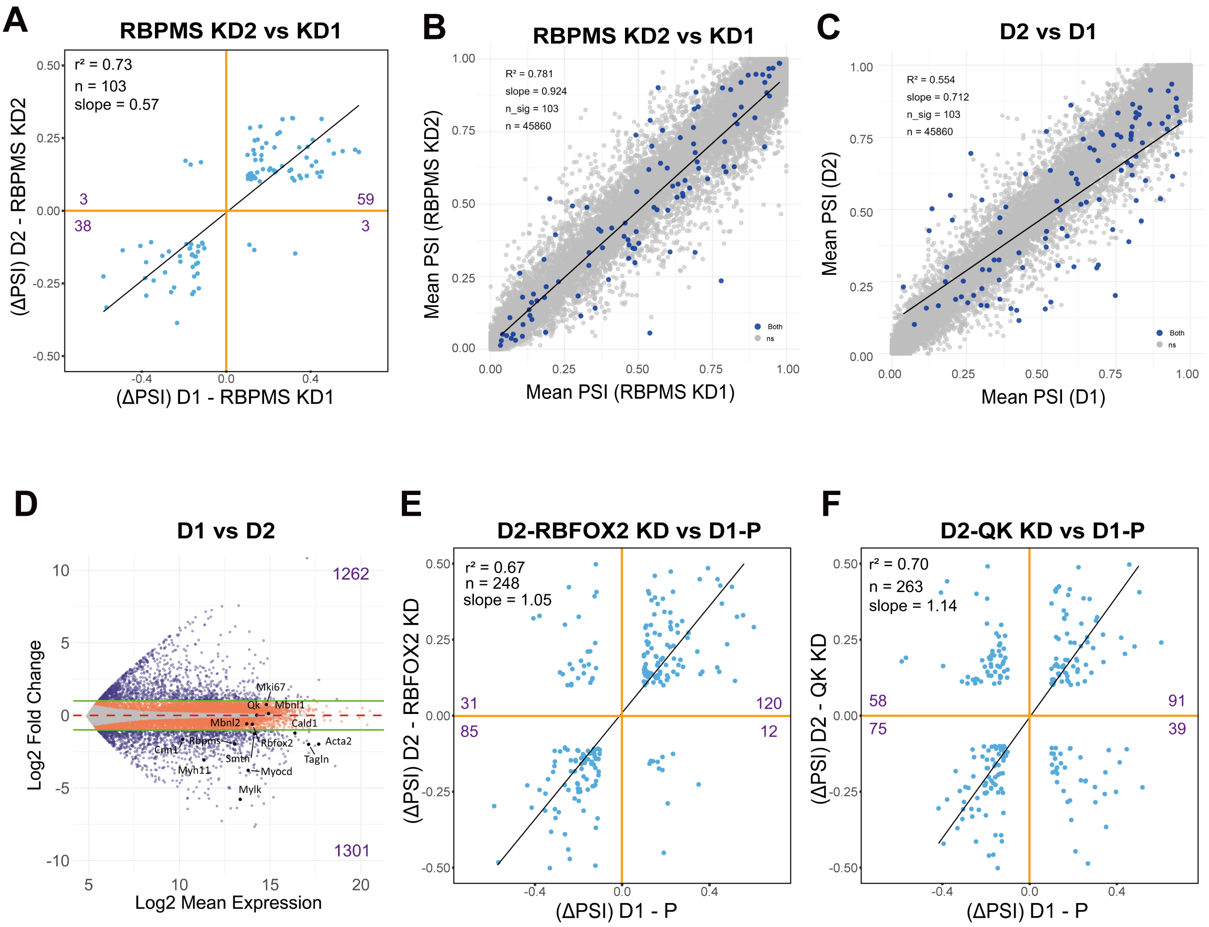

Supplementary Figure S2. Comparative analysis of splicing and RNA abundance across RBPMS knockdowns and differentiation states.

(A) Scatter plot comparing ΔPSI values of RBPMS KD1 (experiment 1) versus RBPMS KD2 (experiment 2) for 103 overlapping alternative splicing events. Pearson correlation coefficient (r^2), slope, and quadrant counts are shown. **(B, C)** Scatter plots comparing mean PSI values between (B) RBPMS KD1 and KD2, and (C) D1 and D2 PAC1 cells. Each point represents one alternative splicing event. Events significant in both datasets are highlighted in blue, while all events are shown in grey. Pearson correlation coefficient (r^2) and slope are calculated for blue data points. **(D)** MA plot showing differential gene expression between D1 and D2 PAC1 cells. Significantly upregulated and downregulated genes (adjusted $p < 0.05$) are shown in colour. Selected SMC marker genes are annotated. Horizontal lines indicate $\log_2$ fold changes of ±1. Purple numbers indicate the counts of significantly upregulated and downregulated genes. **(E-F)** Scatter plots comparing ΔPSI values from (E) D1-P vs D2-RBFOX2 KD and (F) D1-P vs D2-QK KD. Pearson correlation statistics and linear regression slopes are shown. Numbers of alternative splicing events in each quadrant are indicated in purple.

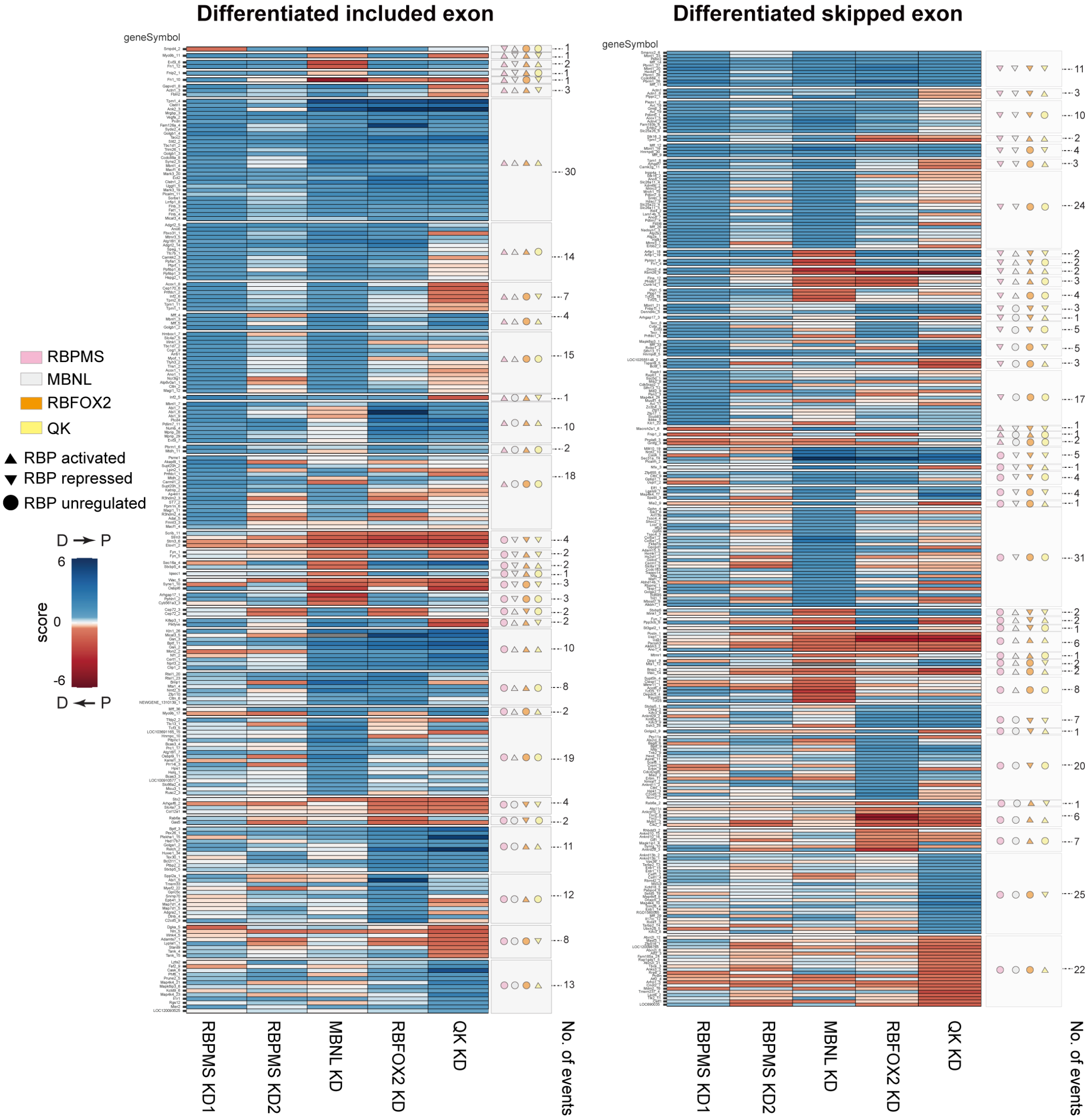

Supplementary Figure S3. Co-regulation of all alternative splicing event types by four RBPs.

Heatmaps showing the effects of individual RBP knockdowns on alternative splicing events that are significantly more included (left) or more skipped (right) in differentiated (D) versus proliferative (P) PAC1 cells. Events include cassette exons (SE), retained introns (RI), mutually exclusive exons (MXE), and alternative 3' and 5' splice site (A3SS and A5SS) types. For each RBP knockdown condition, the ΔPSI change was normalised to the D vs P ΔPSI from RNA-seq experiment 1. Colours indicate the concordance (blue) or antagonism (red) of RBP activity with the D vs P splicing program. Annotation symbols to the right indicate inferred activity of each RBP: activated (▲), repressed (▼), or unregulated (●). The number on the right of the heatmap indicates the counts of events in each cluster.

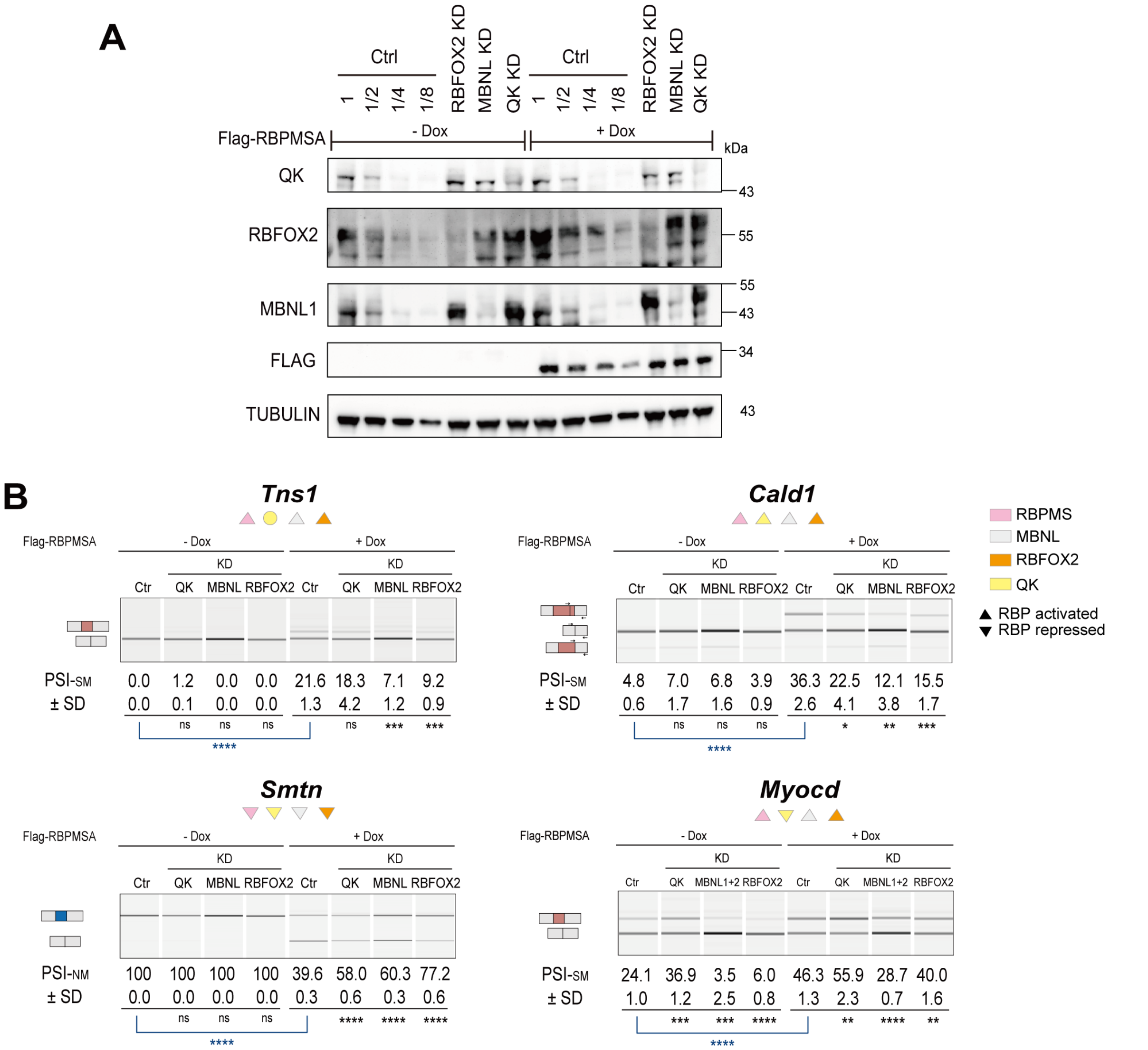

Supplementary Figure S4. RBFOX2, MBNL1/2 and QK act concordantly with pInducer overexpressed RBPMS in PAC1-P cells.

(A) Western-blot validation of inducible Flag-RBPMSA over-expression in PAC1 P cells cultured in the absence (-Dox) or presence (+Dox) of doxycycline, and of MBNL, RBFOX2 and QK knockdown. Immunoblots show both Flag-RBPMSA induction and knock-down efficiency of QK, RBFOX2 and MBNL1. Tubulin served as a loading control. Protein molecular masses (kDa) are indicated on the right. Lanes 1-4, 8-11 have a titration of control samples to allow assessment of knockdown efficiency. **(B)** RT-PCR analysis of alternative splicing events (ASEs). Each panel reports PSI values (mean ± SD) for *Tns1, Cald1, Smtn and Myocd*. Schematic gel images depict the RT-PCR products corresponding to the differentiated (pink) or proliferative (blue) isoforms. Note that in *Cald1* the SM isoform involves inclusion of exon 4 and use of a downstream 5' splice site on exon 3. PCR involved a reverse primer in exon 5 and two forward primers in exon 3 to capture use of the two alternative 5' splice sites; the two forward primers are

positioned such that the longer exon 3 isoform produces a shorter amplicon. siRNA knock-down was performed in the absence (-Dox) or presence (+Dox) of Flag-RBPMSA expression. Blue lines indicate comparisons between the two control lanes. Statistical significance was assessed by two-way ANOVA ($p < 0.05$ *, < 0.01 **, < 0.001 ***, < 0.0001 ****; ns, not significant; NA, not applicable). Arrowheads above each graph denote inferred activity of each RBP: activation (▲), repression (▼), unregulated (●).

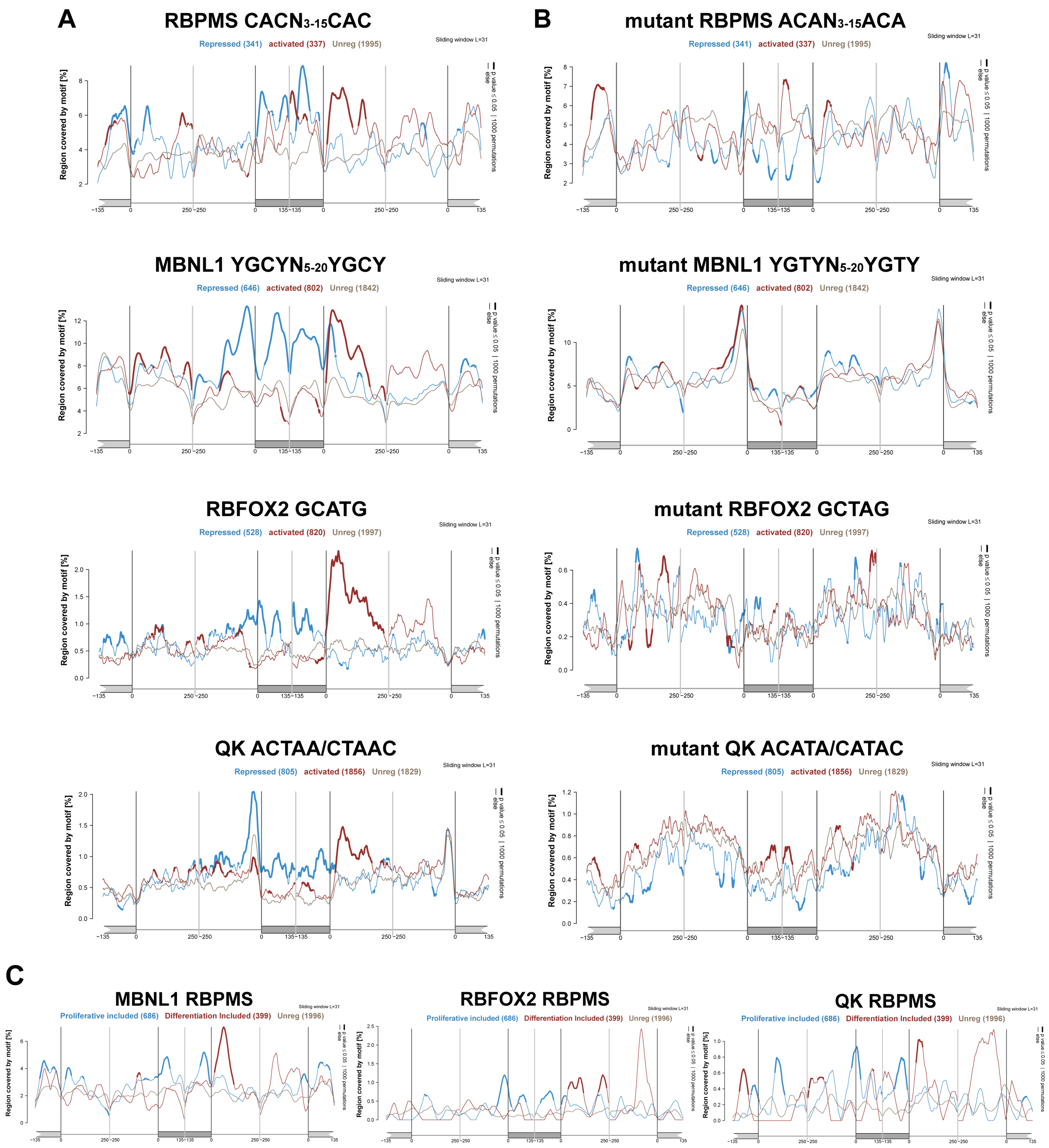

Supplementary Figure S5. RNA motif enrichment in RBP knockdown-regulated exons and co-enrichment of upstream RBP motifs with downstream RBPMS motifs during PAC1 dedifferentiation.

(A) RNA maps showing individual motif enrichment for RBPMS, MBNL, RBFOX2, and QK in cassette exons regulated by their own knockdown. Events were classified as either repressed (blue) or activated (red) by each RBP (JC ≥ 50, |ΔPSI| ≥ 5%, FDR < 0.05), and compared to a randomly downsampled background of unregulated exons (grey; |ΔPSI| < 5%, FDR > 0.1; JC ≥ 50). **(B)** Corresponding enrichment maps using mutated versions of each RBP motif. Motif mutations include: RBPMS (ACAN$_{3-15}$ACA), MBNL (YGTYN$_{5-20}$YGTY), RBFOX2 (GCTAG), and QK (ACATA or CATAC). **(C)** Linked motif enrichment maps showing upstream MBNL, RBFOX2, or QK motifs followed by

downstream RBPMS motifs (within 5-25 nt). Linked motifs were defined as: MBNL (YGCY-N_{5-20}-YGCY), RBFOX2 (GCATG), or QK (ACTAA/CTAAC), upstream of RBPMS (CAC-N_{3-15}-CAC). This is the reverse orientation of Figure 5B-D, where RBPMS motifs were upstream. Motif enrichment in all maps was calculated using the MATT toolkit with a 31-nucleotide sliding window. The y-axis indicates the percentage of nucleotides within each window that are covered by the motif. The x-axis spans ±250 nt of the flanking introns and ±135 nt of the alternative exon and adjacent constitutive exons. Statistically significant occurrences were determined by permutation testing (1,000 iterations, $P \leq 0.05$).

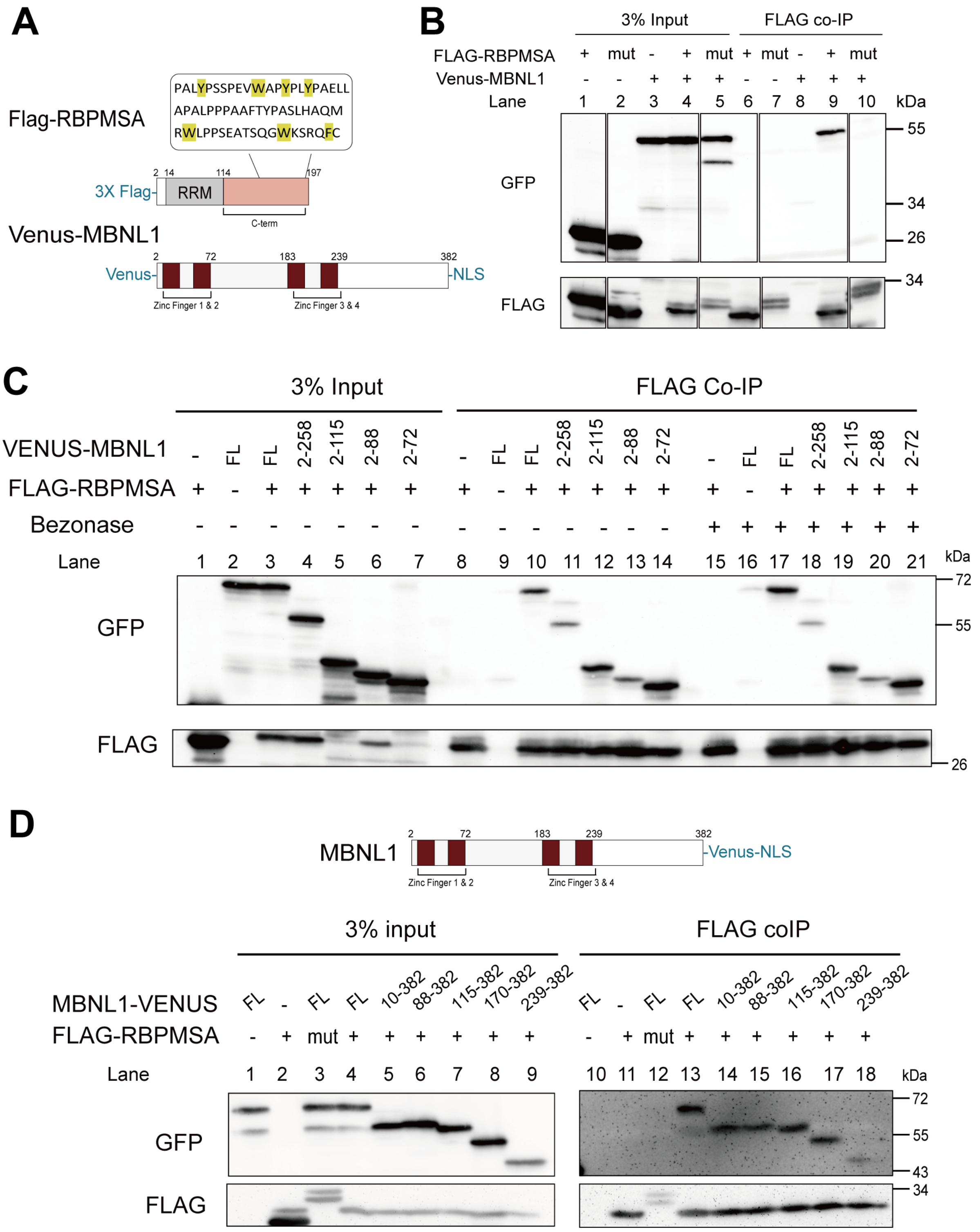

Supplementary Figure S6. RBPMSA interacts with MBNL1 through multiple regions, and the aromatic residues of RBPMSA are required for this interaction.

(A) Schematic representation of the RBPMSA and MBNL1 constructs used in co-immunoprecipitation (co-IP) assays. The aromatic residues of the RBPMSA mutant and the four zinc finger domains of MBNL1, are indicated. **(B)** Co-IP of wild-type or aromatic-residue-mutant (mut) FLAG-RBPMSA with Venus-MBNL1. HEK293T cells were co-transfected with the indicated constructs, and FLAG immunoprecipitation was

performed. Input (3%) and co-IP samples were analysed by western blotting using anti-GFP and anti-FLAG antibodies. The RBPMSA mutant also serves as a negative control. **(C)** Co-IP mapping using a series of Venus-MBNL1 N-terminal truncation mutants (residues 2-72, 2-88, 2-115, 2-258, 2-382(FL)) co-expressed with FLAG-RBPMSA. FLAG immunoprecipitates and 3% input were analysed using anti-GFP and anti-FLAG antibodies. **(D)** Reciprocal mapping co-IP analysis using Venus-tagged C-terminal deletion mutants of MBNL1 (Δ10-382, Δ88-382, Δ115-382, Δ170-382, Δ239-382) co-expressed with FLAG-RBPMSA (WT or mut). Input (3%) and FLAG co-IP samples were analysed by western blotting using anti-GFP and anti-FLAG antibodies. All co-IP experiments were repeated independently three times. Molecular weight markers (kDa) are indicated on the right of each blot.

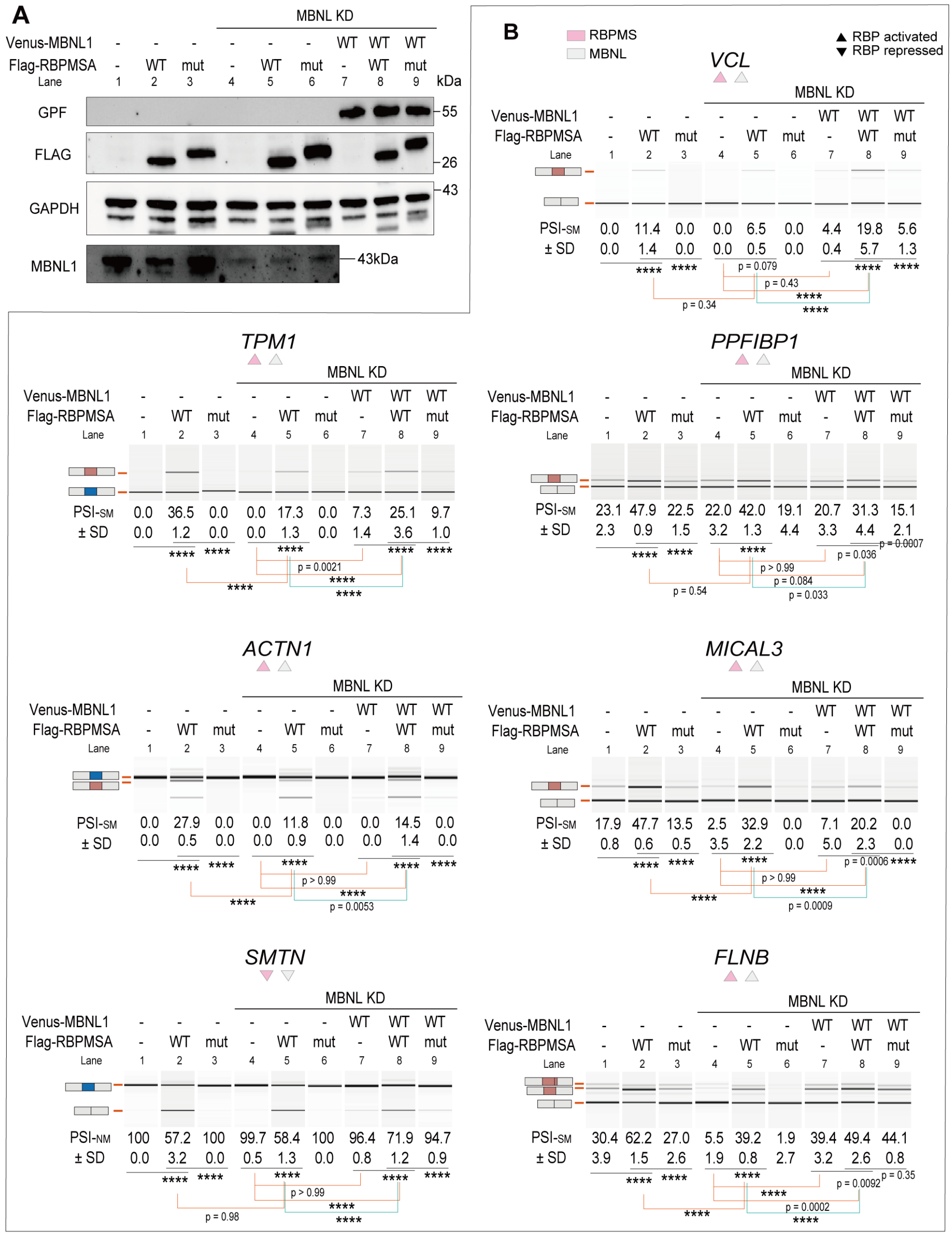

Supplementary Figure S7. RBPMSA and MBNL1 co-regulate SM-associated splicing events.

(A) Western blot validation of MBNL1 knockdown and overexpression conditions used for the RT-PCR assays in (B). HEK293T cells were transfected with Venus-MBNL1 (wild-type, WT) and/or FLAG-RBPMSA (WT or aromatic-residue mutant, mut) under control or MBNL1-knockdown (KD) conditions. Blots were probed with anti-GFP, anti-FLAG, anti-MBNL1, and anti-GAPDH antibodies. GAPDH serves as a loading control. **(B)** RT-PCR analysis of representative SM-associated splicing events under the indicated combinations of MBNL1+2 KD (MBNL KD) and RBPMSA/MBNL1 expression. Genes analysed include VCL, TPM1, ACTN1, PPFIBP1, MICAL3, SMTN, and FLNB. PSI values for SM or NM isoforms are shown below each gel (mean ± SD). Key comparisons: lanes 1 vs

2 evaluate RBPMSA expression; 2 vs 3 the effect of RBPMSA mutation; 4 vs 5 test RBPMSA add-back after MBNL1 loss; 4 vs 7 test MBNL1 complementation; and 4/7 vs 8 assess combined add-back (MBNL1 + RBPMSA). Statistical significance was determined by two-way ANOVA with multiple comparisons; ns and *, **, *** p-values are indicated on the graphs, and **** denotes $p < 0.0001$. Error bars represent mean ± SD. Experiments were repeated independently three times. Coloured triangles above each panel denote inferred RBP function, with activation (▲) or repression (▼) behaviour determined from PAC1 knockdown data.

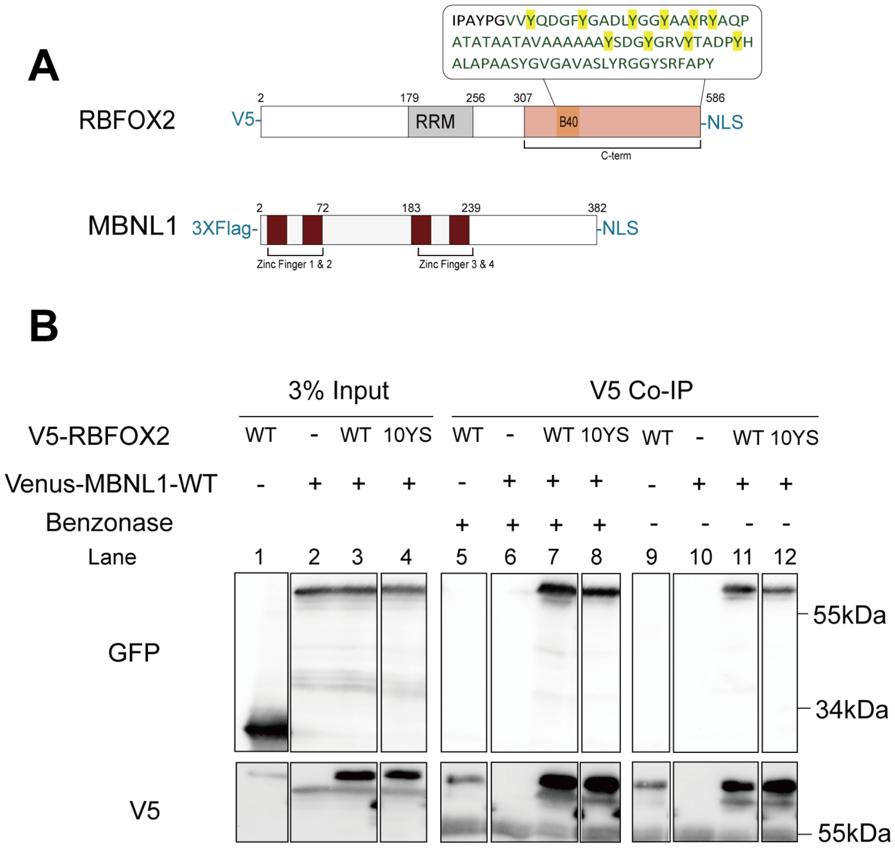

Supplementary Figure S8. RBFOX2 interacts with MBNL1 independently of its Y-region.

(A) Schematic diagrams of V5-tagged RBFOX2 and 3×FLAG-tagged MBNL1 constructs used in co-IP assays. Epitope tags and key functional domains are indicated. Tyrosine residue mutations in the RBFOX2 10YS mutant are highlighted in yellow. **(B)** Co-IP of V5-RBFOX2 (wild-type, WT; or Y-region mutant, 10YS) with Venus-MBNL1. Input (3%) and V5 co-IP samples were analysed by western blotting using anti-GFP and anti-V5 antibodies. Benzonase treatment was included where indicated to assess RNA dependence of the interaction. Molecular weight markers (kDa) are indicated on the right.

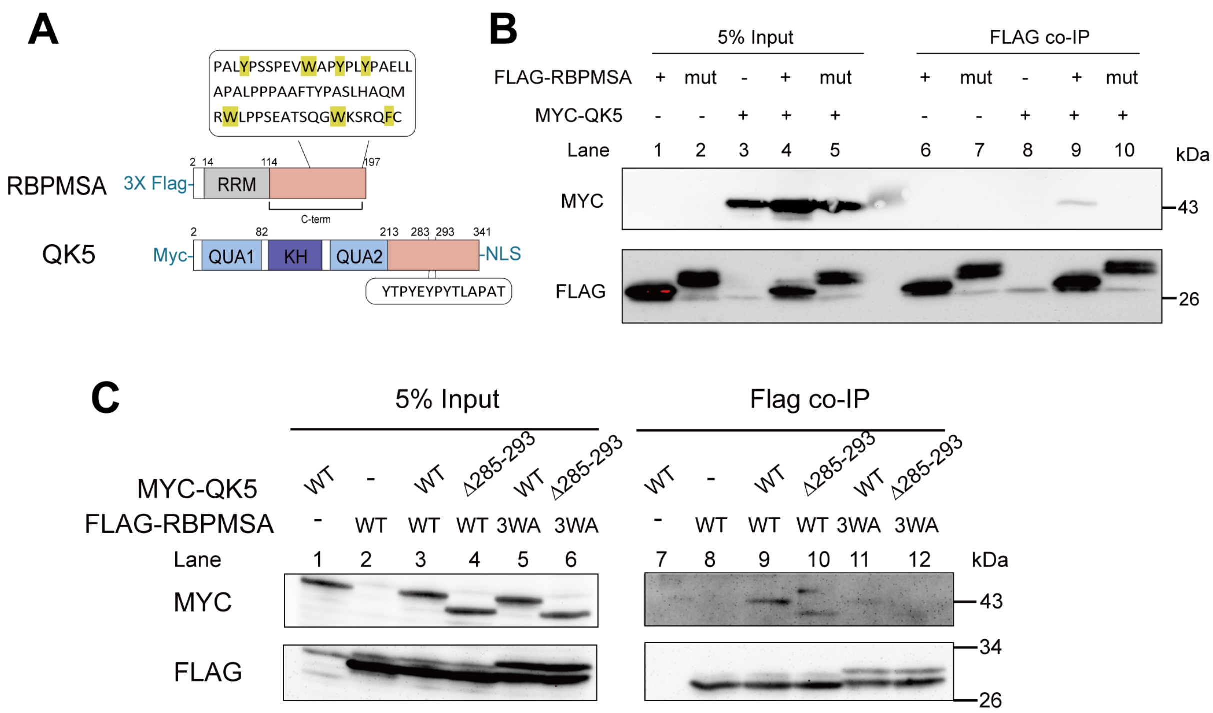

Supplementary Figure S9. Aromatic residues of RBPMSA are required for the interaction with QK5.

(A) Schematic diagrams of FLAG-RBPMSA and MYC-QK5 constructs used in co-IP assays. Epitope tags and key functional domains are indicated. Aromatic residues mutated to alanine in the RBPMSA mutant are highlighted in yellow, and the QK5 C-terminal YTPYEPPYTLAPAT motif deleted in the Δ285-293 mutant is shown. **(B)** Co-IP of FLAG-RBPMSA (wild-type, WT; or aromatic-residue mutant, mut) with MYC-QK5. HEK293T cells were co-transfected with the indicated constructs, and FLAG pull-downs were analysed by western blotting with anti-MYC and anti-FLAG antibodies. Input (5%) and FLAG co-IP samples are shown. **(C)** Co-IP analysis of FLAG-RBPMSA (WT or 3WA mutant (the highlighted three tryptophan in (A) mutated to alanine)) with MYC-QK5 WT or the deletion mutant (Δ285-293). Input (5%) and FLAG co-IP samples were analysed by western blotting using anti-MYC and anti-FLAG antibodies. All co-IP experiments were repeated independently three times. Molecular-weight markers (kDa) are indicated on the right of each blot.

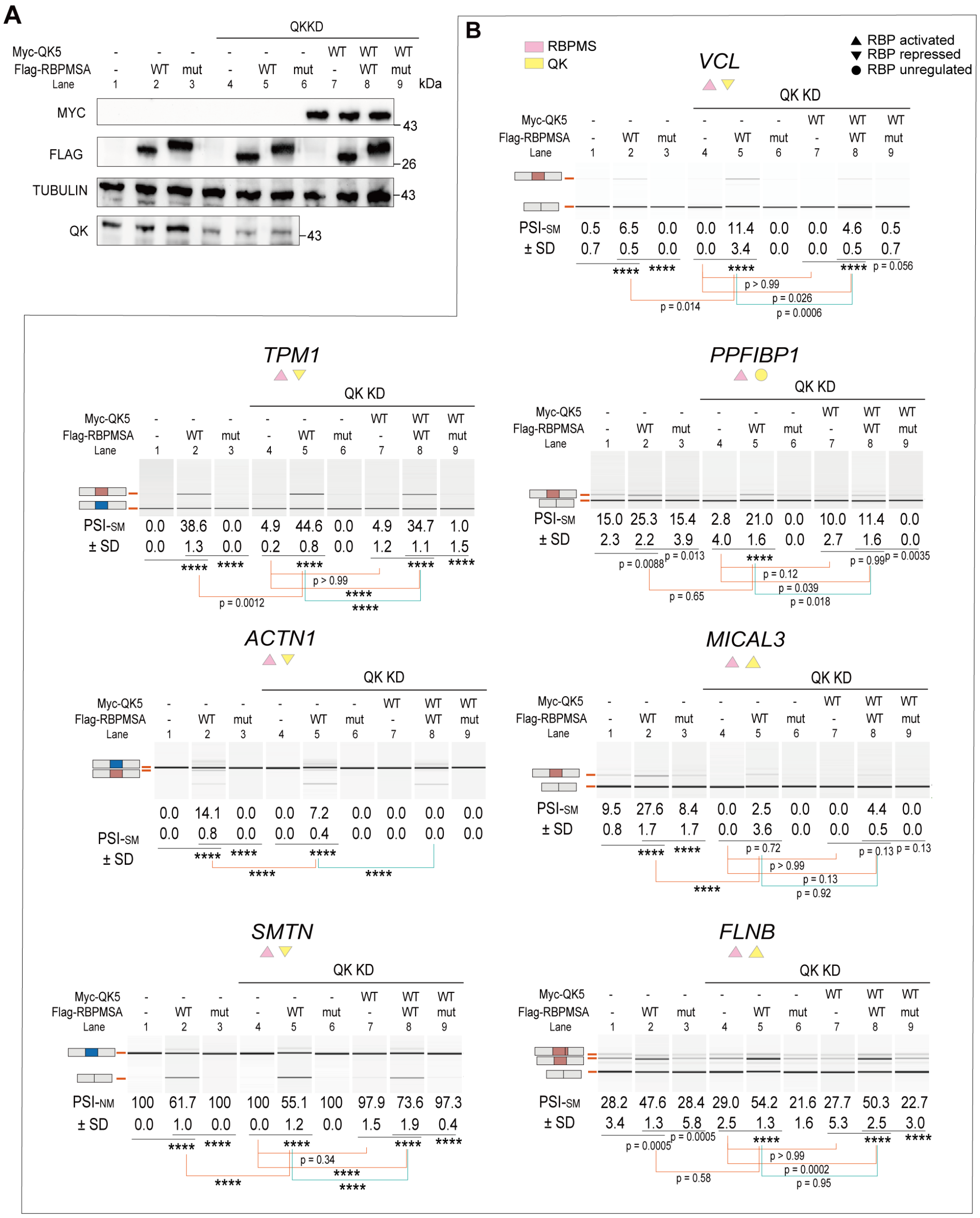

Supplementary Figure S10. RBPMSA and QK5 co-regulate SM-associated splicing events.

(A) Western blot validation of QK5 knockdown and overexpression conditions used for the RT-PCR assays in (B). HEK293T cells were transfected with MYC-QK5 (wild-type, WT) and/or FLAG-RBPMSA (WT or aromatic-residue mutant, mut) under control or QK5-knockdown (QK KD) conditions. Blots were probed with anti-MYC, anti-FLAG, anti-QK, and anti-TUBULIN antibodies. TUBULIN serves as a loading control. **(B)** RT-PCR analysis of representative SM-associated splicing events under the indicated combinations of QK5 KD and RBPMSA/QK5 expression. Genes analysed include *VCL*, *TPM1*, *ACTN1*, *PPFIBP1*, *MICAL3*, *SMTN*, and *FLNB*. PSI values for PSI-SM or PSI-NM isoforms are shown below each gel (mean ± SD). Statistical significance was determined by two-way

ANOVA with multiple comparisons; ns and *, **, *** p-values are indicated on the graphs, and **** denotes $p < 0.0001$. Error bars represent mean ± SD. Data shown are from one representative experiment performed in biological triplicate. Arrowheads above each graph indicate inferred RBP activity: activation (▲), repression (▼), or unregulated (●), based on their behaviour in PAC1 knockdown experiments.

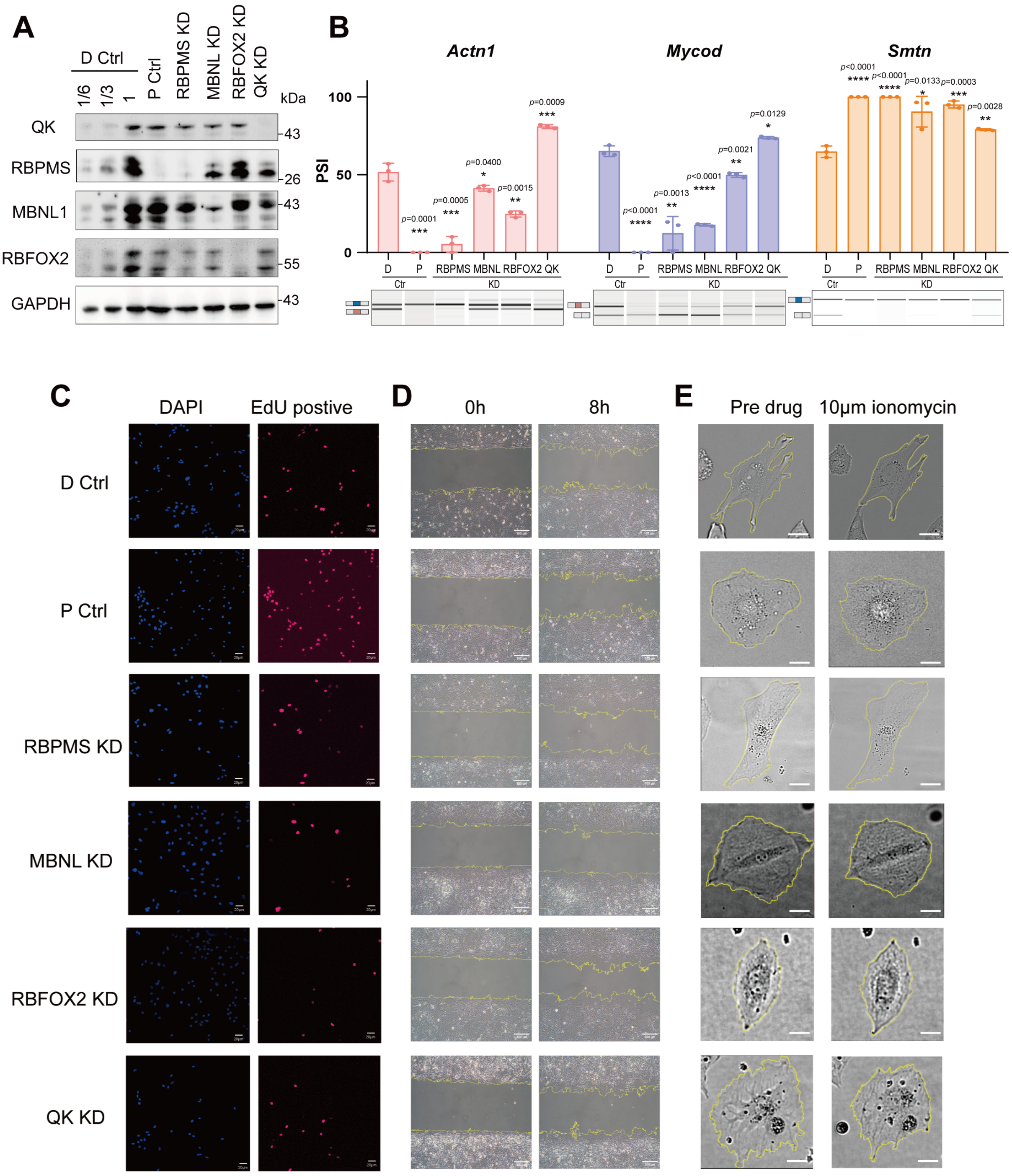

Supplementary Figure S11. Validation of RBP knockdown and representative images for functional assays.

(A) Western blot analysis confirming efficient knockdown of QK, RBPMS, MBNL1+2 (MBNL), and RBFOX2 in differentiated PAC1 cells. GAPDH was used as a loading control. Protein molecular weights (kDa) are indicated. The D Ctrl lane includes serial dilutions (1, 1/3, 1/6) for semi-quantitative comparison. **(B)** RT-PCR analysis of alternative splicing events in *Actn1, Myocd*, and *Smtn* confirming the regulatory effects

of RBPMS, MBNL1+2 (MBNL), RBFOX2, or QK knockdown in differentiated PAC1 cells. Bar graphs show PSI (percent spliced-in) values (mean ± SD, $n = 3$). Schematic diagrams represent alternative isoforms, with NM (non-muscle) exons shown in blue and SM (smooth muscle) exons in pink. Below each graph are representative Qiaxcel gel traces. Statistical comparisons were performed between differentiated control and knockdown conditions using unpaired two-tailed *t*-tests (* $p < 0.05$, p < 0.01, *** $p < 0.001$, **** p < 0.0001). **(C)** Representative EdU staining images for conditions shown in Figure 7A. DAPI (blue) marks nuclei, and EdU (pink) marks replicating cells. Scale bars, 20 μm. **(D)** Representative images from the wound assay shown in Figure 7B, captured at 0 h and 8 h. Yellow lines indicate wound edges used for quantification. Scale bars, 100 μm. **(E)** Representative images from contraction assay shown in Figure 7C. Images were taken before (Pre drug) and after treatment with 10 μM ionomycin. Cell outlines were traced and area contraction quantified. Scale bars, 20 μm.
